# Effect of different types of regular exercise on physical fitness in adults with overweight or obesity: Systematic review and meta‐analyses

**DOI:** 10.1111/obr.13239

**Published:** 2021-05-03

**Authors:** Marleen A. van Baak, Adriyan Pramono, Francesca Battista, Kristine Beaulieu, John E. Blundell, Luca Busetto, Eliana V. Carraça, Dror Dicker, Jorge Encantado, Andrea Ermolao, Nathalie Farpour‐Lambert, Euan Woodward, Alice Bellicha, Jean‐Michel Oppert

**Affiliations:** ^1^ NUTRIM School of Nutrition and Translational Research in Metabolism, Department of Human Biology Maastricht University Medical Centre+ Maastricht The Netherlands; ^2^ Sport and Exercise Medicine Division, Department of Medicine University of Padova Padova Italy; ^3^ Appetite Control and Energy Balance Group (ACEB), School of Psychology, Faculty of Medicine and Health University of Leeds Leeds UK; ^4^ Department of Medicine University of Padova Padova Italy; ^5^ European Association for the Study of Obesity (EASO), Obesity Management Task Force (OMTF) London UK; ^6^ Faculdade de Educação Física e Desporto CIDEFES, Universidade Lusófona de Humanidades e Tecnologias Lisbon Portugal; ^7^ Department of Internal Medicine, Hasharon Hospital, Rabin Medical Center, Sackler School of Medicine Tel Aviv University Tel Aviv Israel; ^8^ APPsyCI – Applied Psychology Research Center Capabilities & Inclusion ISPA – University Institute Lisbon Portugal; ^9^ Obesity Prevention and Care Program Contrepoids. Service of Endocrinology, Diabetology, Nutrition and Patient Education, Department of Internal Medicine University Hospitals of Geneva and University of Geneva Geneva Switzerland; ^10^ Nutrition and Obesities: Systemic Approaches, NutriOmics Sorbonne University, INSERM Paris France; ^11^ University Paris‐Est Créteil, UFR SESS‐STAPS Créteil France; ^12^ Assistance Publique‐Hôpitaux de Paris (AP‐HP), Pitié‐Salpêtrière Hospital, Department of Nutrition, Institute of Cardiometabolism and Nutrition Sorbonne University Paris France

**Keywords:** exercise modality, maximal oxygen uptake, muscle strength

## Abstract

This systematic review examined the effect of exercise training interventions on physical fitness in adults with overweight or obesity and compared the effectiveness of different types of exercise training. Four electronic databases were searched. Articles were included if they described randomized controlled trials of exercise training interventions and their effect on maximal oxygen consumption or muscle strength in adults with overweight or obesity. Changes in outcome parameters were analyzed using random effects meta‐analyses for different training types (aerobic, resistance, combined aerobic plus resistance, and high‐intensity interval training). Eighty‐eight articles satisfied the inclusion criteria of which 66 (3964 participants) could be included in the meta‐analyses. All training types increased VO_2max_ (mean difference 3.82 ml/min/kg (95% CI 3.17, 4.48), *P* < 0.00001; *I* ^2^ = 48%). In direct comparisons, resistance training was less effective in improving VO_2max_ than aerobic training, HIIT was slightly more effective than aerobic training, and no difference between aerobic and combined aerobic plus resistance training was found. For muscle strength benefits, incorporation of resistance exercise in the training program is indicated. Exercise training increases VO_2max_ and muscle strength in adults with overweight or obesity. Differences between training types should be weighed with other needs and preferences when health professionals advise on exercise training to improve physical fitness.

## 1 INTRODUCTION

In the general population, regular exercise is well‐known to have beneficial effects on physical fitness, such as an increase in aerobic capacity and increased muscle strength, which are important for the ability to lead a physically active life and for health.^1^ In order to define the role of regular exercise in the management of adults with overweight or obesity, it is important to know what the effects on physical fitness are in this specific group of individuals. In general, physical fitness (per kg body weight) is lower in individuals with than in those without overweight or obesity.^2^, ^3^ We therefore started with a literature search of systematic reviews on this topic that were published over the last 10 years (2009–2019). This resulted in six reviews.^4^, ^5^, ^6^, ^7^, ^8^, ^9^

Miller et al.^4^ reviewed the effects of different modalities of exercise training during diet‐induced weight loss interventions in adults with obesity. Fourteen randomized controlled trials (RCT's) were included. The authors concluded that exercise training during energy restriction improved strength and cardiovascular fitness. No clear conclusion about the differences between training modalities was drawn. The review by Baker et al.^5^ addressed the effects of physical activity interventions specifically in postmenopausal women with overweight or obesity. The review included five large RCT's (with multiple publications) with long duration (≥12 months). The inclusion criteria of the review were not very strict (some normal‐weight women were included; intervention and control groups differed not only for physical activity). The authors concluded that physical activity interventions have a positive impact on physical capacity. Different types of exercise were included, but differences were not evaluated. Batacan et al.^6^ focused on the effects of high intensity interval training (HIIT). Sixty‐five studies with different study designs in populations with normal weight and overweight were included. The inclusion criteria were not strict: in many studies categorized as studies in individuals with overweight or obesity, this was not mentioned as one of the inclusion criteria in the original study. The authors concluded that short (<12 weeks) and long‐term (≥12 weeks) HIIT improve VO_2max_ in individuals with overweight or obesity as well as in individuals in the non‐overweight group.

Türk et al.^8^ compared the effects of high intensity (continuous and interval) exercise and lower intensity endurance exercise or no exercise on VO_2max_. Fifteen studies were included. The conclusion of the authors was that high intensity exercise was superior to improve cardiopulmonary fitness in comparison with lower intensity or no exercise in adults with obesity. Su et al.^9^ compared the effects of HIIT with those of moderate intensity continuous training (MICT). Sixteen studies were included. Overall HIIT was equally effective as MICT in improving VO_2max_, but HIIT training with longer (≥2 min) intervals appeared to be more effective than MICT. Hita‐Contreras et al.^7^ reviewed the effects of exercise interventions on muscle strength and gait speed in sarcopenic obesity. Five RCT's were included, two of which used electrostimulation as exercise intervention. The authors concluded that exercise increases grip strength and gait speed in people with sarcopenic obesity. The effects of different types of training were not analyzed.

Because of the limited information available from these systematic reviews, which mostly focused on specific groups, did not distinguish between types of training or studied the combination with a weight loss diet, we decided to do a systematic review and meta‐analyses on the topic in the context of the EASO Physical Activity Working Group guidelines development. The aim of this systematic review was to examine the impact of various types of exercise interventions (aerobic training, resistance training, combined aerobic and resistance training, and high‐intensity interval training) on physical fitness, with a focus on cardiorespiratory fitness and muscle strength, in adults with overweight or obesity and compare their effectiveness. A secondary aim was to compare the effectiveness in adults with normal weight and those with overweight or obesity.

## 2 METHODS

This systematic review follows the Preferred Reporting Items for Systematic Reviews and Meta‐Analysis (PRISMA) guidelines and is registered in the PROSPERO database (registration number CRD42019157823).

### 2.1 Search strategy

Four electronic databases (PubMed, Web of Science, Cochrane Library, and EMBASE) were searched for original RCTs published up to December 2019. The specific key words used for the search are listed in Table S1. Reference lists from the resulting articles were screened to identify additional articles.

### 2.2 Study selection, inclusion, and exclusion

Articles were included if the RCT involved adults (>18 years, no maximum age) with overweight (BMI ≥ 25 kg/m^2^) or obesity (BMI ≥ 30 kg/m^2^) participating in physical activity interventions, that is, exercise training programs, or interventions promoting increases in physical activity. Studies focusing on the primary prevention of weight gain/obesity were not included. The presence of obesity comorbidities, such as type 2 diabetes, hypertension, dyslipidemia, metabolic syndrome, liver disease (NAFLD/NASH), and osteoarthritis, was not an exclusion criterion. Exercise training programs included regular sessions with one or more types of exercise (aerobic and/or resistance and/or high‐intensity interval training). Exercise sessions could be supervised, partially supervised or non‐supervised. Exercise interventions in combination with other interventions (e.g., diet) were excluded. Comparators included no intervention, another form of exercise or sham exercise (e.g., stretching).

Abstracts and full texts were assessed for eligibility independently by two authors (M. A. v. B. and A. P.) with uncertainty regarding eligibility discussed among authors.

### 2.3 Data extraction

Data were extracted by two authors using standardized forms. The following characteristics were extracted: reference, study design, number of participants included in intervention and control groups, population characteristics (age, BMI, %female, comorbidities for intervention, and control groups), description of intervention (program duration, number of sessions per week, type of training/topic, and supervision/delivery) and comparison, and outcomes. Additional data were obtained from six authors.^10^, ^11^, ^12^, ^13^, ^14^, ^15^

The findings pertaining to cardiorespiratory fitness (measured or estimated VO_2max_), muscle strength, and any other parameter of physical function of each included article are reported.

### 2.4 Data synthesis and statistical analysis

To calculate the effect size of each study, we used the mean change (post‐ to pre‐intervention) and SD of the variable of interest over the experimental period in the control and intervention groups. If these values were not reported, we calculated the mean difference as the difference in mean pre and post intervention and its SD using the formula: SD = square root ((SD_pretreatment_)^2^ + (SD_posttreatment_)^2^) − (2r × SD_pretreatment_ × SD_posttreatment_)). Because the pretest–posttest correlation coefficients (*r*) were not reported in the studies, a conservative *r* value of 0.5 was assumed throughout. If an exact p value for the within group intervention effect was reported, we used this value together with the subject number (*N*) to estimate the SD of the effect.

The meta‐analysis of included studies was conducted using the Cochrane Review Manager 5.3 software.^16^ If a study included more than two experimental groups, which were compared with one control group, the number of subjects in the control group was divided by the number of comparisons. The effect size was expressed as mean difference (MD) or standardized mean difference (SMD) when not all studies reported the outcome in the same units. Most studies reported data from a completers analysis. In a limited number of studies intention‐to‐treat analysis was performed and, if available, these results were included in the meta‐analyses.

Random‐effects models were used for the statistical analysis. The effect size is reported as the (standardized) mean difference with its 95% confidence interval (95% CI). Effect sizes were considered large, medium, small and negligible when SMD was >0.8, between 0.5 and 0.8, between 0.2 and 0.5 and below 0.2, respectively.^17^ Heterogeneity was measured using the *I* ^2^ test.^18^ Heterogeneity was considered low, moderate, and high when *I* ^2^ was <50%, between 50 and 75% and ≥75%.^19^

### 2.5 Quality assessment

Study quality was assessed with a standardized tool including 14 criteria, as previously described.^20^ Study quality was defined as good, fair, or poor when 0, 1, or ≥2 criteria defined as “fatal flaws” (randomization, dropout <20%, intention‐to‐treat analysis) were not fulfilled. Quality assessment was conducted independently by two reviewers (M. A. v. B. and A. P.) using this standardized tool. Any disagreement between the reviewers was resolved through discussion.

### 2.6 Risk of bias assessment

Publication bias was assessed by visual inspection of the funnel plots. When the funnel plot showed signs of asymmetry and the number of included studies was >10, Egger's test was performed.

## 3 RESULTS

The database search yielded 3068 articles, 2831 of which were eliminated based on titles and abstracts alone. Twenty‐five studies were added from additional sources. Full texts were retrieved from 162 articles and 88 satisfied the inclusion criteria (Figure S1). Of these, 66 could be included in the various meta‐analyses. The 66 studies included 3954 participants, sample size ranged from 12 to 464 (median 43), training duration ranged from 2 to 70 weeks (median 12 weeks), % females participating in the studies ranged from 0 to 100% (median 82%), mean age in study groups ranged between 20 and 75 years (median 46 y), mean baseline BMI ranged between 26.4 and 37.2 kg/m^2^ (median 30.9) and mean baseline VO_2max_ ranged between 14.9 and 39.7 ml/min/kg (median 28.4). The quality assessment yielded 21 studies that were of good quality, 28 of fair and 17 of poor quality, mainly due to high dropout rates and lack of an intention‐to‐treat analysis (Table S2). Table 1 summarizes the effects of different types of exercise training on selected physical fitness parameters in adults with overweight or obesity.

**TABLE 1:** Summary of meta‐analyses on the effects of different types of exercise training (aerobic, resistance, combined aerobic plus resistance, and high‐intensity interval training) on VO_2max_, muscle strength and other fitness parameters in adults with overweight or obesity

Outcome	Intervention	N	Comparator	N	(S)md	95% CI		*I*^2^ (%)	*P* value
						min	max		
VO_2_max	Aerobic	995	No exercise	447	4.08	3.22	4.95	61	0.00001
	Resistance	75	No exercise	84	4.52	1.76	7.28	59	0.001
	Aerobic + Resistance	165	No exercise	153	4.57	2.14	7.00	74	0.0002
	HIIT	183	No exercise	123	4.31	2.81	5.80	51	<0.00001
	Resistance	143	Aerobic	137	−1.40	−2.41	−0.38	11	0.007
	Aerobic + Resistance	96	Aerobic	97	0.38	−0.63	1.38	0	0.46
	HIIT	300	Aerobic	221	0.99	0.25	1.73	0	0.008
Muscle strength	Aerobic	78	No exercise	76	0.26	−0.06	0.58	0	0.12
	Resistance	291	No exercise	206	0.74	0.54	0.93	0	<0.00001
	Aerobic + Resistance	74	No exercise	71	0.62	0.27	0.96	0	0.004
	Resistance	96	Aerobic	95	0.49	0.19	0.78	0	0.001
Physical fitness (flexibility, balance, walking speed, and global physical capacity score)	Resistance or Aerobic + Resistance	131	No exercise	98	0.66	0.37	0.95	0	<0.00001

*Note*: For muscle strength, no studies on the effects of HIIT were found.

Abbreviations: CI, confidence interval; HIIT, high‐intensity interval training; *I* ^2^, heterogeneity; MD, mean difference; *N*, number of participants; SMD, standardized mean difference; VO_2_max, maximum oxygen consumption.

### 3.1 Effect of exercise training on cardiorespiratory fitness

For the meta‐analyses on cardiorespiratory fitness the outcome was maximal or peak oxygen consumption (VO_2max_). Only studies which expressed VO_2max_ in ml/min/kg were included to take the effect of potential weight changes during the intervention period into account.

#### 3.1.1 Effect of aerobic exercise training on cardiorespiratory fitness

The characteristics of the 24 included RCTs^10^, ^11^, ^14^, ^21^, ^22^, ^23^, ^24^, ^25^, ^26^, ^27^, ^28^, ^29^, ^30^, ^31^, ^32^, ^33^, ^34^, ^35^, ^36^, ^37^, ^38^, ^39^, ^40^, ^41^ with 1802 participants are presented in Table S3. The meta‐analysis had 41 study arms with 995 individuals in the experimental groups and 447 in the control groups. It showed that aerobic exercise training significantly improved VO_2max_ (mean difference (MD) 4.08 ml/min/kg (95% CI 3.22, 4.95), *P* < 0.00001). The heterogeneity was moderate (*I* ^2^ = 61%). The effect size was medium (standardized mean difference (SMD) 0.70 (95% CI 0.58, 0.82)). Figure S2 shows the forest plot. One of the factors that may have influenced the heterogeneity among studies was the duration of the intervention. Studies were subdivided in three groups according to intervention duration: <12 weeks (eight studies, *N* = 244), 12 weeks (11 studies, *N* = 268) and >12 weeks (seven studies, *N* = 932). In the subgroup with study duration <12 weeks the MD was 3.31 ml/min/kg (95% CI 1.78, 4.83), *P* < 0.0001, *I* ^2^ = 0%. In the subgroup with an intervention duration of 12 weeks the MD was 5.41 ml/min/kg (95% CI 3.88, 6.93), *P* < 0.00001, *I* ^2^ = 39%. When study duration was >12 weeks the MD was 3.34 ml/min/kg (95% CI 2.30, 4.39), *P* < 0.00001, *I* ^2^ = 62%. The heterogeneity remained high in the studies with a duration >12 weeks (13 weeks to 16 months), which may be suggestive for problems with adherence with longer intervention durations. Quality assessment of the studies included in the meta‐analysis showed that 15 of the studies had fair or good quality,^10^, ^14^, ^21^, ^22^, ^23^, ^24^, ^27^, ^29^, ^31^, ^32^, ^35^, ^37^, ^39^, ^40^, ^41^ whereas nine were of poor quality^11^, ^25^, ^26^, ^28^, ^30^, ^33^, ^34^, ^36^, ^38^ (Table S2). Removing the poor‐quality studies from the meta‐analysis slightly reduced the MD in VO_2max_ change to 3.89 ml/min/kg (95% CI 2.79, 5.00), *P* < 0.00001, *I* ^2^ = 65% (*N* = 999). The effect size was medium (SMD 0.66 (95% CI 0.51, 0.80).

The funnel plot looked asymmetric, which was confirmed by Egger's test (*P* = 0.001) (Figure S3). When the study by Church et al.^24^ was excluded, Egger's test was no longer significant (*P* = 0.084). The study by Church et al. had by far the largest number of subjects, had a relatively long duration of 6 months and reported a relatively small effect on VO_2max_ compared to most of the other studies. The participants exercised three times per week 20, 40, or 60 min at 50% VO_2peak_. Despite its size, the study seemed well‐controlled, and no serious compliance issues were reported. This might suggest a small study effect, that is, smaller studies with no significant effect may not have been published. On the other hand, the baseline VO_2peak_ was the lowest of all studies included, which may suggest that it was underestimated, and thus, training intensity was also lower than suggested.

#### 3.1.2 Effect of resistance exercise training on cardiorespiratory fitness

The characteristics of the six included RCTs^14^, ^21^, ^29^, ^33^, ^42^, ^43^ with a total sample size of 195 are presented in Table S4. The meta‐analysis had six study arms with 75 individuals in the experimental groups and 84 in the control groups. It showed that resistance training significantly improved VO_2max_ (MD 4.52 ml/min/kg (95% CI 1.56, 7.28), *P* = 0.001). The heterogeneity was moderate (*I* ^2^ = 59%) (Figure S4). The effect size was large (SMD 0.81 (95% CI 0.22, 1.41)). Quality assessment of the studies included in this meta‐analysis showed that all studies except one^33^ were of fair or good quality (Table S2). Removing this study from the meta‐analysis resulted in an MD of 4.33 ml/min/kg (95% CI 1.11, 7.56), *P* = 0.008, *I* ^2^ = 67%. The funnel plot (Figure S5) showed no evidence of asymmetry.

#### 3.1.3 Effect of combined aerobic plus resistance exercise training on cardiorespiratory fitness

The characteristics of the seven included RCTs^12^, ^14^, ^29^, ^44^, ^45^, ^46^, ^47^ with 377 participants are presented in Table S5. Seven study arms were included with 165 individuals in the experimental groups and 153 in the control groups. The meta‐analysis showed that combined aerobic plus resistance training significantly improved VO_2max_ (MD 4.57 ml/min/kg (95% CI 2.14, 7.00), *P* = 0.0002). The effect size was medium (SMD 0.78 (95% CI 0.40, 1.16)). The heterogeneity was also large (*I* ^2^ = 74%), which was due to the study by Hara et al.^45^ When this study was excluded the MD was reduced to 2.95 ml/min/kg (95% CI 2.05, 3.85), *P* < 0.00001, *I* ^2^ = 0%. The forest plot is shown in Figure S6. The majority of the studies included in this meta‐analysis was of fair‐to‐good quality, only one had poor quality^47^ (Table S2). Excluding this poor‐quality study resulted in an MD of 4.98 ml/min/kg (95% CI 2.07, 7.88), *P* = 0.0008, *I* ^2^ = 78%. The funnel plot looked asymmetric due to the study by Hara et al.^45^ (Figure S7). No Egger's test was performed due to the small number of studies. Removing the study by Hara et al.^45^ diminished the visual asymmetry.

#### 3.1.4 Effect of high intensity interval training (HIIT) on cardiorespiratory fitness

The characteristics of the 10 included RCTs^11^, ^28^, ^31^, ^41^, ^44^, ^48^, ^49^, ^50^, ^51^, ^52^ with a total of 402 participants are presented in Table S6. Sixteen study arms were included with 183 individuals in the experimental groups and 123 in the control groups. The meta‐analysis showed that HIIT significantly improved VO_2max_ (MD 4.31 ml/min/kg (95% CI 2.81, 5.80), *P* < 0.00001). The heterogeneity was moderate (*I* ^2^ = 51%). The effect size was large (SMD 0.84 (95% CI 0.57, 1.11)). The forest plot is shown in Figure S8. Two studies^50^, ^52^ had a very short intervention duration of 3–4 weeks. If these studies were excluded from the meta‐analysis the MD in VO_2max_ increased to 5.29 ml/min/kg (95% CI 3.67, 6.91) (*P* < 0.00001). *I* ^2^ was 30%. The effect size was large (SMD 0.86 (95% CI 0.53, 1.19). Quality assessment of the studies showed that seven of the studies had fair or good quality, whereas three were of poor quality^11^, ^28^, ^48^ (Table S2). When the poor‐quality studies and studies with duration ≤4 weeks were excluded, the MD was 4.82 ml/min/kg (95% CI 2.89, 6.75), *P* < 0.00001, *I* ^2^ = 0%. Visual inspection of the funnel plot (Figure S9) showed some evidence of asymmetry, but Egger's test was not significant (*P* = 0.222).

#### 3.1.5 Comparison of the effect of different types of training on cardiorespiratory fitness

We also analyzed whether a certain training type(s) should be preferred as training regimen for the improvement of VO_2max_ in adult with overweight or obesity. The number of studies that compared certain types of training is limited. We were able to analyze the comparison of aerobic endurance type training with resistance training, with combined aerobic and resistance training and with HIIT.

##### Aerobic training versus resistance training

The characteristics of the eight included RCTs^13^, ^14^, ^15^, ^21^, ^29^, ^33^, ^53^, ^54^ with a total of 322 participants are presented in Table S7. Eight study arms were included with 143 individuals in the resistance training groups and 137 in the aerobic training groups. The meta‐analysis showed that aerobic training improved VO_2max_ more than resistance training (MD −1.40 ml/min/kg (95% CI −2.41, −0.38, *P* = 0.007) (Figure S10). The effect size was small (SMD −0.37 (95% CI −0.63, −0.12)). The heterogeneity was low (*I* ^2^ = 11%). Quality assessment of the studies showed that five of the studies had fair or good quality, whereas three were of poor quality^15^, ^33^, ^53^ (Table S2). After removing the poor‐quality studies from the meta‐analysis the difference in VO_2max_ change was no longer significant (MD −0.65 ml/min/kg (95% CI −2.18, 0.89), *P* = 0.41, *I* ^2^ = 7% (*N* = 133)). The SMD was −0.21 (95% CI −0.55, 0.13) (*P* = 0.23). Visual inspection of the funnel plot (Figure S11) showed some evidence of asymmetry, but the number of studies was too small to perform Egger's test. Leaving out the study by Schroeder et al.^14^ on the bottom left of the funnel plot hardly affected the effect size (MD = −1.45 ml/min/kg (95% CI −2.32, −0.57), *P* = 0.001, *I* ^2^ = 0%).

##### Aerobic training versus combined aerobic plus resistance training

Only four studies were available for this comparison. The characteristics of the four included RCTs^14^, ^15^, ^29^, ^55^ with a total of 221 participants are presented in Table S8. Four study arms were included with 96 individuals in the aerobic plus resistance training groups and 97 in the aerobic training groups. The meta‐analysis showed that there was no statistically significant difference in effect between the two types of training (MD 0.38 ml/min/kg (95% CI −0.63, 1.38), *P* = 0.46) (Figure S12). The SMD was 0.07 (95% CI −0.22, 0.35)). The heterogeneity was low (*I* ^2^ = 0%). Quality assessment of the studies included in the meta‐analysis showed that three of the studies had fair or good quality, whereas one was of poor quality^15^ (Table S2). Removing the poor‐quality study from the meta‐analysis hardly changed the results (MD 0.33 (95% CI −1.38, 2.04), *P* = 0.71, *I* ^2^ = 0%, *N* = 104). Because of the small number of studies, the funnel plot (Figure S13) was not informative.

##### Aerobic training versus high‐intensity interval training

The characteristics of the 18 included RCTs^11^, ^13^, ^28^, ^31^, ^56^, ^57^, ^58^, ^59^, ^60^, ^61^, ^62^, ^63^, ^64^, ^65^, ^66^, ^67^ with a total of 623 participants are presented in Table S9. Twenty‐six study arms were included with 300 individuals in the HIIT groups and 221 in the aerobic training groups. The meta‐analysis showed a small, but statistically significant, difference in effect between the two types of training in favour of HIIT (MD 0.99 ml/min/kg (95% CI 0.25, 1.73), *P* = 0.008). The heterogeneity was low (*I* ^2^ = 0%) (Figure S14). The effect size was small, SMD = 0.20 (95% CI 0.02, 0.38) (*P* = 0.03). When studies with a duration <6 weeks were excluded from the analysis,^58^, ^61^, ^66^ the MD was 1.19 (95% CI 0.41, 1.97), *P* = 0.003, *I* ^2^ = 0%. Quality assessment of the studies included in the meta‐analysis showed that three of the studies had poor quality^11^, ^28^, ^63^ (Table S2). Removing the poor‐quality studies from the meta‐analysis resulted in an MD of 1.02 ml/min/kg (95% CI 0.12, 1.91), *P* = 0.03, *I* ^2^ = 7%, *N* = 407). The funnel plot (Figure S15) showed no evidence of asymmetry (Egger's test *P* = 0.489).

##### Effect of exercise training on VO_2max_ in individuals with or without overweight or obesity

One of the questions we tried to address was whether the effects of training on VO_2max_ are comparable in individuals with or without overweight or obesity. The literature search yielded only two studies,^68^, ^69^ in which a direct comparison between groups with normal weight and with overweight/obesity were made. In the study by Blake et al.^68^ the effects of a 14‐week aerobic training intervention were compared in women with or without overweight or obesity (*N* = 89). The increase in VO_2max_ was comparable in the two groups. In the study by Gondim et al.^69^ the effects of a 12‐month aerobic training intervention were compared in men and women with normal weight, overweight, or obesity (*N* = 143). VO_2max_ (estimated from a 12‐min walk/jog test) was increased compared to baseline at 12 months in the group with normal weight and at 6 months in the group with overweight. No significant VO_2max_ changes were found in the group with obesity. However, the changes over time were not compared among the groups. Clearly more studies are needed to answer the question whether training effects on VO_2max_ are comparable in individuals in different BMI categories.

### 3.2 Effect of exercise training on muscle strength

For the meta‐analyses on muscle strength various outcomes were used, including static or dynamic muscle strength of different muscle groups. When available, strength parameters expressed per kg body weight or muscle mass were included to take the effect of potential weight changes during the intervention period into account. The outcomes of the meta‐analyses are reported as standardized mean differences (SMD), because study outcomes (strength measurements and units of measurement) differed across studies.

#### 3.2.1 Effect of aerobic exercise training on muscle strength

The characteristics of the six included RCTs^14^, ^23^, ^33^, ^37^, ^70^, ^71^ with a total of 206 participants are presented in Table S10. Twelve study arms were included with 78 individuals in the aerobic exercise groups and 76 in the no exercise control groups. The meta‐analysis showed no significant difference in effect (SMD 0.26 (95% CI −0.06, 0.58), *P* = 0.12). The heterogeneity was low (*I* ^2^ = 0%) (Figure S16). Quality assessment of the studies included in the meta‐analysis showed that two of the six studies had poor quality^33^, ^70^ (Table S2). Removing the poor‐quality studies from the meta‐analysis resulted in a SMD of 0.08 (95% CI −0.41, 0.57), *P* = 0.75, *I* ^2^ = 0%, *N* = 66). Visual inspection of the funnel plot (Figure S17) did not suggest asymmetry.

#### 3.2.2 Effect of resistance exercise training on muscle strength

The characteristics of the 12 included RCTs^14^, ^33^, ^42^, ^70^, ^71^, ^72^, ^73^, ^74^, ^75^, ^76^, ^77^, ^78^ with 612 participants are presented in Table S11. Thirty‐two study arms were included with 291 individuals in the resistance exercise groups and 206 in the no exercise control groups. The meta‐analysis showed a significant difference in effect in favor of resistance training (SMD 0.74 (95% CI 0.54, 0.93), *P* < 0.00001). The heterogeneity was low (*I* ^2^ = 0%) (Figure S18). Quality assessment of the studies included in the meta‐analysis showed that four of the 12 studies had poor quality^33^, ^70^, ^71^, ^77^ (Table S2). Removing the poor‐quality studies from the meta‐analysis resulted in a SMD of 0.76 (95% CI 0.53, 0.98), *P* < 0.00001, *I* ^2^ = 0%, *N* = 379). The funnel plot (Figure S19) showed no evidence of asymmetry (Egger's test *P* = 0.698).

#### 3.2.3 Effect of combined aerobic and resistance exercise training on muscle strength

The characteristics of the six included RCTs^14^, ^46^, ^47^, ^70^, ^79^, ^80^ with a total of 218 participants are presented in Table S12. Thirteen study arms were included with 74 individuals in the aerobic plus resistance exercise groups and 71 in the no exercise control groups. The meta‐analysis also showed a significant difference in effect (standardized mean difference (SMD) 0.62 (95% CI 0.27, 0.96), *P* = 0.0004). The heterogeneity was low (*I* ^2^ = 0%) (Figure S20). Quality assessment of the studies included in the meta‐analysis showed that two of the six studies had poor quality^47^, ^70^ (Table S2). Removing the poor‐quality studies from the meta‐analysis resulted in a SMD of 0.67 (95% CI 0.24, 1.10), *P* = 0.002, *I* ^2^ = 0%, *N* = 93). Visual inspection of the funnel plot did not suggest serious asymmetry (Figure S21).

#### 3.2.4 Effect of high‐intensity interval training on muscle strength

There were no studies that investigated the effect of HIIT on muscle strength in comparison to no exercise training.

#### 3.2.5 Comparison of the effect of different types of training on muscle strength

##### Resistance training versus aerobic training

The characteristics of the seven included RCTs^13^, ^33^, ^53^, ^70^, ^71^, ^81^ with 251 participants are presented in Table S13. Fourteen study arms were included with 96 individuals in the resistance exercise groups and 95 in the aerobic exercise groups. The meta‐analysis showed a significant difference in effect in favour of resistance training (SMD 0.49 (95% CI 0.19, 0.78), *P* = 0.001). The heterogeneity was low (*I* ^2^ = 0%) (Figure S22). Quality assessment of the studies included in the meta‐analysis showed that four of the seven studies had poor quality^33^, ^53^, ^70^, ^71^ (Table S2). Removing the poor‐quality studies from the meta‐analysis resulted in a SMD of 0.46 (95% CI −0.01, 0.92), *P* = 0.06, *I* ^2^ = 0%, *N* = 74). The funnel plot (Figure S23) showed some evidence of asymmetry with studies missing at the right‐hand side of the plot, but due to the small number of studies Egger's test was not performed.

##### Resistance training versus combined aerobic plus resistance training

Three studies additionally compared the effects of combined aerobic plus resistance training with aerobic training^14^, ^15^, ^70^ (data not shown). No significant difference was found: SMD 0.50 (95% CI −0.21, 1.21), *P* = 0.16, *I* ^2^ = 70%. The study by Slentz et al.^15^ was a clear outlier. Removing this study resulted in a SMD 0.17 (95% CI −0.33, 0.67), *P* = 0.50, *I* ^2^ = 0%.

##### Resistance training versus HIIT

No studies.

#### 3.2.6 Effect of exercise training on muscle strength in individuals with or without overweight or obesity

Four studies^68^, ^77^, ^82^, ^83^ were found that directly compared the effects of exercise training on muscle strength in individuals with normal weight and with overweight or obesity. Pescatello et al.^82^ and Vincent et al.^77^ studied the effects of resistance training and found no difference in the strength response between the group with normal weight and that with overweight or obesity. On the other hand, no differences in the strength or flexibility response to combined aerobic and resistance exercise training were found by Blake et al.^68^ Gondim et al.^69^ reported an increase in muscle strength in the group with obesity, but not in the group with normal weight, however whether there was a significant difference between the groups was not reported.

### 3.3 Exercise training and other physical fitness parameters

Six studies^46^, ^72^, ^73^, ^76^, ^79^, ^80^ reporting the effect of resistance or combined aerobic and resistance exercise training on muscle strength also provided data on the effects on other parameters of physical fitness, such as flexibility, balance, global physical capacity score, walking speed.

The meta‐analysis (Figure S24) showed that resistance training and combined aerobic plus resistance exercise training had a positive effect on these parameters (SMD 0.66 (95% CI 0.37, 0.95), *P* < 0.00001). Heterogeneity was low (*I* ^2^ = 0%). Visual inspection of the funnel plot (Figure S25) did not suggest asymmetry.

Three studies^23^, ^37^, ^38^ reported on the effect of aerobic training on flexibility. Two^23^, ^37^ found an improvement, one no effect.^38^ Manini et al.^83^ compared the effect of a combination of different training modalities (aerobic, resistance, and flexibility) on gait speed and Short Physical Performance Battery (SPPB) in individuals with normal weight and with obesity. The responses were less pronounced in the group with obesity.

## 4 DISCUSSION

In this systematic review and series of meta‐analyses we tried to determine the effect of different training modalities on cardiorespiratory fitness and muscle strength in individuals with overweight or obesity. Additionally, we investigated the effects on other parameters of physical fitness (e.g., overall score, flexibility, balance, walking speed). We also tried to evaluate whether the responses were similar in groups with normal weight and groups with overweight or obesity. Our main findings are that all included training modalities (aerobic, resistance, combined aerobic and resistance and forms of high‐intensity interval training) improve cardiorespiratory fitness, as measured by maximal oxygen uptake per kg body weight. In direct comparisons, resistance training was less effective than aerobic training and there was no difference between combined aerobic plus resistance training and aerobic training alone. HIIT had a slightly larger effect on VO_2max_ than aerobic training. With respect to muscle strength, resistance training and combined aerobic and resistance training increased muscle strength, whereas aerobic training did not. No data were available for HIIT. In a direct comparison, resistance training was more effective in improving muscle strength than aerobic training. Other comparisons could not be made because of lack of data. Limited evidence was found that resistance and combined aerobic plus resistance training also improve other aspects of physical fitness such as flexibility, balance, walking speed, and overall physical fitness score.

Physical fitness is important for the tasks of everyday life and health and this review shows that individuals with overweight or obesity can increase aspects of their physical fitness by participating in exercise training programs without focus on weight loss, with effect sizes ranging from medium to large. The question whether the responses are similar in individuals with normal weight and those with overweight or obesity could not be answered based on the studies included in this systematic review.

In general, this systematic review and the included meta‐analyses confirm the results of previous ones examining the effects of exercise training on physical fitness in (subgroups of) adults with overweight or obesity,^4^, ^5^, ^6^, ^7^, ^8^, ^9^ but extends these by using more strict inclusion criteria and a more detailed comparison among training modalities. Aerobic training, as expected, increased VO_2max_ as did combined aerobic plus resistance training. Whether resistance training also improves VO_2max_ is a topic of discussion in the literature.^84^, ^85^ We found that resistance training was also effective, although less than aerobic training, in increasing VO_2max_, which may be related to the relatively low level of baseline VO_2max_ in the population with overweight or obesity.^84^

The majority of studies in this review were in line with general recommendations for aerobic and resistance exercise for the improvement or maintenance of physical fitness (e.g., that of the American College of Sports Medicine^86^), although the combination of the two, which is recommended in most guidelines, was studied less frequently. We found that combined aerobic and resistance exercise was equally effective as aerobic training in improving VO_2max_, whereas aerobic plus resistance training was equally effective as resistance training for muscle strength, although this latter conclusion was based on only three studies.

Studies included males and females with a wide range of mean ages (18–75 years), BMIs (26.4–40.5 kg/m^2^) and initial fitness levels (VO_2max_ 14.9–39.5 ml/min/kg) and therefore appear to cover the overall adult population with overweight or obesity, except for the morbid obesity category. Nevertheless, not all training modalities were studied in the same populations. For instance, participants in the high‐intensity training studies were generally younger, whereas those participating in training studies where muscle strength was the outcome were on average older and had lower BMIs.

Intervention durations ranged between 2 and 70 weeks. There was some evidence that very short (2–4 weeks) and very long durations (>26 weeks) were associated with less pronounced training effects, which may be related with the time course of obtaining the optimal training effect: in short studies the optimal effect may not have been attained yet, whereas in longer duration interventions reduced compliance may play a role in diminishing the training effect.

In conclusion, in individuals with overweight or obesity all training modalities included in this review (aerobic, resistance, combined aerobic and resistance and high‐intensity interval training) increased VO_2max_. HIIT and exercise programs that included aerobic training were the most effective. For muscle strength benefits, incorporation of resistance exercise in the training program is required. Health professionals should weigh these differences with the needs and preferences of the individual with overweight or obesity when advising on exercise.

## CONFLICT OF INTEREST

No conflict of interest statement.

## Supporting information

**Table S1.** Keywords included in database search strategy
**Table S2**. Quality assessment of the studies included in the meta‐analyses.
**Table S3**. Characteristics of the randomised controlled trials included in the meta‐analysis on the effect of aerobic exercise training on VO2max.
**Table S4**. Characteristics of the randomised controlled trials included in the meta‐analysis on the effect of resistance exercise training on VO2max.
**Table S5**. Characteristics of the randomised controlled trials included in the meta‐analysis on the effect of combined aerobic and resistance exercise training on VO2max.
**Table S6**. Characteristics of the randomised controlled trials included in the meta‐analysis on the effect of high‐intensity interval training on VO2max.
**Table S7**. Characteristics of the randomised controlled trials included in the meta‐analysis comparing the effects of aerobic and resistance training on VO2max in adults with overweight or obesity.
**Table S8**. Characteristics of the randomised controlled trials included in the meta‐analysis comparing the effects of aerobic and combined aerobic plus resistance training on VO2max in adults with overweight or obesity.
**Table S9**. Characteristics of the randomised controlled trials included in the meta‐analysis comparing the effects of aerobic and high‐intensity interval (HIIT) training on VO2max in adults with overweight or obesity.
**Table S10**. Characteristics of the randomised controlled trials included in the meta‐analysis on the effects of aerobic training on muscle strength in adults with overweight or obesity.
**Table S11**. Characteristics of the randomised controlled trials included in the meta‐analysis on the effects of resistance training on muscle strength in adults with overweight or obesity.
**Table S12**. Characteristics of the randomised controlled trials included in the meta‐analysis on the effects of combined aerobic plus resistance training on muscle strength in adults with overweight or obesity.
**Table S13**. Characteristics of the randomised controlled trials included in the meta‐analysis comparing the effects of resistance training and aerobic training on muscle strength in adults with overweight or obesity.
**Figure S1**. Flow chart of literature search.
**Figure S2**. Forest plot of the effect of aerobic training vs no training on VO2max in adults with overweight or obesity.
**Figure S3**. Funnel plot of the effect of aerobic endurance training on VO2max in adults with overweight or obesity.
**Figure S4**. Forest plot of the effect of resistance training vs no training on VO2max in adults with overweight or obesity.
**Figure S5**. Funnel plot of the effect of resistance training vs no training on VO2max in adults with overweight or obesity.
**Figure S6**. Forest plot of the effect of aerobic plus resistance training vs no training on VO2max in adults with overweight or obesity.
**Figure S7**. Funnel plot of the effect of resistance training vs no training on VO2max in adults with overweight or obesity.
**Figure S8**. Forest plot of the effect of high‐intensity interval training (HIIT) vs no training on VO2max in adults with overweight or obesity.
**Figure S9**. Funnel plot of the effect of high‐intensity interval training (HIIT) on VO2max in adults with overweight or obesity.
**Figure S10**. Forest plot comparing the effects of resistance training with aerobic training on VO2max in adults with overweight or obesity.
**Figure S11**. Funnel plot of studies comparing the effects of aerobic and resistance training on VO2max in adults with overweight or obesity.
**Figure S12**. Forest plot comparing the effects of combined aerobic plus resistance training with aerobic training on VO2max in adults with overweight or obesity.
**Figure S13**. Funnel plot of studies comparing the effects of combined aerobic plus resistance training and aerobic training on VO2max in adults with overweight or obesity.
**Figure S14**. Forest plot comparing the effects of HIIT with aerobic training on VO2max in adults with overweight or obesity.
**Figure S15**. Funnel plot of studies comparing the effects of HIIT and aerobic training on VO2max in adults with overweight or obesity.
**Figure S16**. Forest plot comparing the effects of aerobic training vs no training on muscle strength in adults with overweight or obesity.
**Figure S17**. Funnel plot of the effect of aerobic training on muscle strength in adults with overweight or obesity.
**Figure S18**. Forest plot comparing the effects of resistance training vs no training on muscle strength in adults with overweight or obesity.
**Figure S19**. Funnel plot of the effect of resistance training on muscle strength in adults with overweight or obesity.
**Figure S20**. Forest plot comparing the effects of combined aerobic plus resistance training vs no training on muscle strength in adults with overweight or obesity.
**Figure S21**. Funnel plot of the effect of combined aerobic plus resistance training on muscle strength in adults with overweight or obesity.
**Figure S22**. Forest plot comparing the effects of resistance training vs aerobic training on muscle strength in adults with overweight or obesity.
**Figure S23**. Funnel plot of the effect of resistance training vs aerobic training on muscle strength in adults with overweight or obesity.
**Figure S24**. Forest plot of the effects of resistance training alone or in combination with aerobic training on parameters of physical fitness (flexibility, balance, walking speed, global physical capacity score) in adults with overweight or obesity.
**Figure S25**. Funnel plot of the effect of resistance training on parameters of physical capacity in adults with overweight or obesity.
