# Supplementary material for: Effect of different types of regular exercise on physical fitness in adults with overweight or obesity: Systematic review and meta‐analyses
Source: Obes Rev. 2021 May 3;22(Suppl 4):e13239. doi: 10.1111/obr.13239 (PMC8365680; doi:10.1111/obr.13239)
Supplement: Supplementary file 1 — Table S1. Keywords included in database search strategy Table S2. Quality assessment of the studies included in the meta‐analyses. Table S3. Characteristics of the randomised controlled trials included in the meta‐analysis on the effect of aerobic exercise training on VO2max. Table S4. Characteristics of the randomised controlled trials included in the meta‐analysis on the effect of resistance exercise training on VO2max. Table S5. Characteristics of the randomised controlled trials included in the meta‐analysis on the effect of combined aerobic and resistance exercise training on VO2max. Table S6. Characteristics of the randomised controlled trials included in the meta‐analysis on the effect of high‐intensity interval training on VO2max. Table S7. Characteristics of the randomised controlled trials included in the meta‐analysis comparing the effects of aerobic and resistance training on VO2max in adults with overweight or obesity. Table S8. Characteristics of the randomised controlled trials included in the meta‐analysis comparing the effects of aerobic and combined aerobic plus resistance training on VO2max in adults with overweight or obesity. Table S9. Characteristics of the randomised controlled trials included in the meta‐analysis comparing the effects of aerobic and high‐intensity interval (HIIT) training on VO2max in adults with overweight or obesity. Table S10. Characteristics of the randomised controlled trials included in the meta‐analysis on the effects of aerobic training on muscle strength in adults with overweight or obesity. Table S11. Characteristics of the randomised controlled trials included in the meta‐analysis on the effects of resistance training on muscle strength in adults with overweight or obesity. Table S12. Characteristics of the randomised controlled trials included in the meta‐analysis on the effects of combined aerobic plus resistance training on muscle strength in adults with overweight or obesity. Table S13. Characteristics of the r [file OBR-22-e13239-s001.pdf]

SUPPORTING MATERIAL

Effect of different types of regular exercise on physical fitness in adults with overweight or obesity: systematic review and meta-analyses

Marleen A. van Baak[1]*, Adriyan Pramono[1], Francesca Battista[2], Kristine Beaulieu[3], John E. Blundell[3], Luca Busetto[4,5], Eliana V. Carraça[6], Dror Dicker[5,7], Jorge Encantado[8], Andrea Ermolao[2], Nathalie Farpour-Lambert[5,9], Euan Woodward[5], Alice Bellicha[10,11], Jean-Michel Oppert[12]

[1] NUTRIM School of Nutrition and Translational Research in Metabolism, Department of Human Biology, Maastricht University Medical Centre+, Maastricht, The Netherlands

[2] Sport and Exercise Medicine Division, Department of Medicine, University of Padova, Padova, Italy

[3] Appetite Control and Energy Balance Group (ACEB), School of Psychology, Faculty of Medicine and Health, University of Leeds, Leeds, LS2 9JT, UK

[4] Department of Medicine, University of Padova, Padova, Italy

[5] European Association for the Study of obesity (EASO), Obesity Management Task Force (OMTF)

[6] CIDEFES, Universidade Lusófona de Humanidades e Tecnologias, Faculdade de Educação Física e Desporto. Lisboa, Portugal

[7] Department of Internal Medicine D, Hasharon Hospital, Rabin Medical Center, Sackler School of Medicine, Tel Aviv University, Tel Aviv, Israel

[8] APPsyCI – Applied Psychology Research Center Capabilities & Inclusion; ISPA - University Institute

[9] Obesity Prevention and Care Program Contrepoids. Service of Endocrinology, Diabetology, Nutrition and Patient Education, Department of Internal Medicine, University Hospitals of Geneva and University of Geneva, Switzerland

[10] Sorbonne University, INSERM, Nutrition and obesities: systemic approaches, NutriOmics, F-75013, Paris, France;

[11] University Paris-Est Créteil, UFR SESS-STAPS, Créteil, France

[12] Assistance Publique-Hôpitaux de Paris (AP-HP), Pitié-Salpêtrière hospital, Department of Nutrition, Institute of Cardiometabolism and Nutrition; Sorbonne University, France

***Corresponding Author**

Marleen A. van Baak

Department of Human Biology, NUTRIM School for Nutrition and Translational Research in Metabolism, Maastricht University Medical Centre+, Universiteitssingel 50, 6229 ER Maastricht, The Netherlands; Tel: +31 43 3881630; Fax: +31 43 3690976

E-mail: m.vanbaak@maastrichtuniversity.nl

Table S1. Keywords included in database search strategy

Obesity	Physical activity	Age	Topic
Overweight<br>Obesity<br>Obese	Physical activit*<br>Exercise<br>Sport<br>Endurance activity<br>Endurance activities<br>Aerobic activity<br>Aerobic activities<br>Cardiovascular activit*<br>Resistance training<br>Strength training<br>Muscle-strengthening<br>Weight-Lifting program<br>High-intensity interval training<br>HIIT<br>Physical conditioning	Adults<br>(NOT child, children, adolescents, pediatric)<br>Humans	Cardio-respiratory fitness,<br>physical fitness,<br>maximal oxygen uptake,<br>maximal oxygen consumption,<br>musc* fitness,<br>musc* strength,<br>VO_{2max},<br>aerobic capacity,<br>walking distance,<br>anaerobic capacity

Table S2. Quality assessment of the studies included in the meta-analyses.

**Reference**	**Criteria**														**Total "Yes"**	**Total "No"**	**Total "other"**	**Quality rating**
	**1**	**2**	**3**	**4**	**5**	**6**	**7**	**8**	**9**	**10**	**11**	**12**	**13**	**14**				
Ahmadzad 2007[21]	Yes	NR	NR	No	NR	NR	Yes	No	NR	Yes	NR	No	Yes	Yes	5	3	7	FAIR
Arad 2015[48]	Yes	NR	NR	NR	NR	Yes	No	Yes	Yes	Yes	Yes	No	Yes	No	7	3	4	POOR
Baekkerud 2015[56]	Yes	Yes	NR	No	NR	Yes	Yes	No	Yes	Yes	Yes	Yes	Yes	No	9	3	2	FAIR
Banitalebi 2019[44]	Yes	Yes	Yes	No	Yes	Yes	Yes	No	Yes	Yes	Yes	No	Yes	No	10	4	0	FAIR
Batrakoulis 2018[42]	Yes	Yes	Yes	No	NR	NR	Yes	No	Yes	Yes	Yes	Yes	Yes	No	9	3	2	FAIR
Blond 2019[10]	Yes	Yes	Yes	No	No	Yes	No	No	Yes	Yes	Yes	Yes	Yes	Yes	10	4	0	FAIR
Bonfante 2017[47]	Yes	NR	NR	No	NR	Yes	No	CD	Yes	Yes	Yes	No	Yes	No	6	4	4	POOR
Brooker 2019[22]	Yes	Yes	Yes	No	NR	Yes	Yes	No	Yes	Yes	Yes	No	Yes	No	9	4	1	FAIR

Cao 2019[23]	Yes	NR	NR	No	NR	Yes	Yes	Yes	Yes	Yes	Yes	No	Yes	No	8	3	3	FAIR
Cheema 2015[57]	Yes	Yes	Yes	No	NR	Yes	Yes	No	No	No	Yes	No	Yes	Yes	8	5	1	GOOD
Chen 2017[70]	Yes	NR	NR	No	Yes	Yes	No	Yes	NR	Yes	Yes	No	Yes	No	7	4	3	POOR
Chin 2020[11]	Yes	NR	NR	No	NR	Yes	No	No	Yes	Yes	Yes	No	Yes	No	6	5	3	POOR
Church 2007[24]	Yes	Yes	Yes	No	Yes	Yes	Yes	No	Yes	Yes	Yes	Yes	Yes	Yes	12	2	0	GOOD
Cocks 2016[58]	Yes	NR	NR	No	NR	Yes	Yes	Yes	NR	Yes	Yes	Yes	Yes	Yes	9	1	4	GOOD
De Strijcker 2018[59]	Yes	Yes	NR	No	NR	Yes	Yes	No	Yes	Yes	Yes	No	Yes	Yes	9	3	2	GOOD
Donnelly 2013[25]	Yes	Yes	Yes	No	Yes	Yes	No	No	Yes	Yes	Yes	Yes	Yes	No	10	4	0	POOR
Duscha 2005[26]	Yes	Yes	NR	No	NR	Yes	No	NR	Yes	Yes	Yes	No	Yes	No	7	4	3	POOR
Emerenziani 2014[27]	Yes	NR	NR	No	NR	Yes	Yes	No	NR	Yes	Yes	No	Yes	Yes	7	3	4	GOOD
Fisher 2015[60]	Yes	Yes	Yes	No	NR	Yes	Yes	No	NR	Yes	Yes	No	Yes	Yes	9	3	2	GOOD
Fritz 2018[72]	Yes	Yes	Yes	No	NR	Yes	Yes	No	Yes	Yes	Yes	No	Yes	No	9	4	1	FAIR

Gerosa-Neto 2019[28]	Yes	NR	NR	No	NR	Yes	No	No	Yes	Yes	Yes	NR	Yes	No	6	4	4	POOR
Hara 2005[45]	Yes	Yes	Yes	No	NR	Yes	Yes	Yes	NR	Yes	Yes	NR	Yes	Yes	10	1	3	GOOD
Higgins 2016[65]	Yes	NR	NR	No	NR	Yes	Yes	CD	Yes	Yes	Yes	Yes	Yes	No	8	2	4	FAIR
Ho 2012[29]	Yes	Yes	NR	No	NR	Yes	Yes	Yes	NR	Yes	Yes	Yes	Yes	No	9	2	3	FAIR
Irving 2008[30]	Yes	NR	NR	No	NR	Yes	No	Yes	Yes	Yes	Yes	Yes	Yes	No	8	3	3	POOR
Irwin 2003[12]	Yes	Yes	Yes	No	NR	Yes	Yes	Yes	CD	Yes	Yes	No	Yes	Yes	10	2	2	GOOD
Jabbour 2015[49]	Yes	NR	NR	No	NR	Yes	Yes	Yes	NR	Yes	Yes	NR	Yes	Yes	8	1	5	GOOD
Jurio-Iriarte 2018[31]	Yes	NR	NR	No	NR	Yes	Yes	No	NR	Yes	Yes	No	Yes	No	6	4	4	FAIR
Kang 2012[79]	Yes	NR	NR	No	NR	Yes	No	Yes	NR	Yes	Yes	No	Yes	No	6	4	4	FAIR
Keating 2015[32]	Yes	Yes	Yes	No	No	Yes	Yes	Yes	Yes	Yes	Yes	Yes	Yes	Yes	12	2	0	GOOD
Keating 2017[43]	Yes	Yes	Yes	No	No	Yes	Yes	No	Yes	Yes	Yes	No	Yes	Yes	10	4	0	GOOD
Kim 2020[53]	Yes	Yes	Yes	No	NR	Yes	No	Yes	NR	Yes	Yes	No	Yes	No	8	4	2	POOR

Kim 2016[33]	Yes	NR	NR	No	NR	Yes	No	CD	Yes	Yes	Yes	No	Yes	No	6	4	4	POOR
Kirk 2003[34]	Yes	NR	NR	No	NR	Yes	No	NR	Yes	Yes	Yes	NR	Yes	No	6	3	5	POOR
Kong 2016[61]	Yes	NR	NR	No	NR	Yes	Yes	No	NR	Yes	Yes	Yes	Yes	No	7	3	4	FAIR
Liao 2018[73]	Yes	NR	NR	No	Yes	Yes	Yes	Yes	Yes	Yes	Yes	No	Yes	Yes	10	2	2	GOOD
Lunt 2014[62]	Yes	Yes	NR	No	Yes	Yes	No	No	NR	Yes	Yes	Yes	Yes	Yes	9	3	2	FAIR
Martins 2015[63]	Yes	NR	NR	No	NR	Yes	No	No	Yes	Yes	Yes	No	Yes	No	6	5	3	POOR
Moghadasi 2012[35]	Yes	NR	NR	No	NR	Yes	Yes	Yes	NR	Yes	Yes	No	Yes	Yes	8	2	4	GOOD
Nader 2016[36]	No	NR	NR	No	NR	Yes	Yes	Yes	NR	Yes	Yes	NR	Yes	Yes	7	2	5	POOR
Park 2015[46]	Yes	NR	NR	No	NR	Yes	Yes	Yes	NR	Yes	Yes	No	Yes	Yes	8	2	4	GOOD
Park 2017[80]	Yes	NR	NR	No	NR	Yes	Yes	Yes	Yes	Yes	Yes	No	Yes	Yes	9	2	3	GOOD
Plotnikoff 2010[74]	Yes	NR	NR	No	NR	Yes	Yes	Yes	No	Yes	Yes	No	Yes	Yes	8	3	3	GOOD
Ramos 2019[37]	Yes	NR	NR	No	NR	Yes	Yes	Yes	NR	Yes	No	No	Yes	Yes	7	3	4	FAIR

Rayes 2019[38]	No	Yes	Yes	No	NR	Yes	Yes	No	Yes	Yes	Yes	No	Yes	No	8	4	2	POOR
Reichkendler 2014[39]	Yes	NR	NR	No	NR	Yes	Yes	Yes	Yes	Yes	Yes	No	Yes	No	8	3	3	FAIR
Robinson 2015[66]	Yes	NR	NR	No	NR	Yes	Yes	Yes	NR	Yes	Yes	No	Yes	No	7	3	4	FAIR
Rustaden 2017[75]	Yes	Yes	Yes	No	NR	Yes	Yes	No	No	Yes	Yes	Yes	Yes	No	9	3	2	FAIR
Sarsan 2006[71]	Yes	Yes	Yes	No	NR	Yes	No	Yes	NR	Yes	Yes	No	Yes	No	8	4	2	POOR
Sawczyn 2015[54]	Yes	NR	NR	No	NR	Yes	Yes	Yes	NR	Yes	Yes	No	Yes	Yes	8	2	4	GOOD
Sawyer 2016[87]	Yes	NR	NR	No	NR	Yes	Yes	Yes	NR	Yes	Yes	Yes	Yes	No	8	2	4	FAIR
Schjerve 2008[13]	Yes	NR	NR	No	NR	Yes	Yes	Yes	NR	Yes	Yes	No	Yes	Yes	8	2	4	GOOD
Schroeder 2019[14]	Yes	Yes	Yes	No	NR	Yes	Yes	Yes	Yes	Yes	Yes	No	Yes	Yes	11	2	1	GOOD
Skrypnik 2015[55]	Yes	NR	NR	No	NR	Yes	Yes	No	Yes	Yes	Yes	No	Yes	No	7	4	3	FAIR

Slentz 2011[15]	Yes	NR	NR	No	NR	Yes	No	NR	NR	Yes	Yes	No	Yes	No	5	4	5	POOR
Smith-Ryan 2016[50]	Yes	Yes	NR	No	NR	CD	Yes	Yes	NR	Yes	Yes	Yes	Yes	No	8	2	4	FAIR
Sun 2018[64]	Yes	NR	NR	No	NR	Yes	Yes	Yes	NR	Yes	Yes	No	Yes	No	7	3	4	FAIR
Tong 2018[51]	Yes	NR	NR	No	NR	Yes	Yes	Yes	Yes	Yes	Yes	No	Yes	No	8	3	3	FAIR
Trilk 2011[52]	Yes	NR	NR	No	NR	Yes	Yes	Yes	NR	Yes	Yes	Yes	Yes	Yes	9	1	4	GOOD
Utter 1998[40]	Yes	NR	NR	No	NR	Yes	Yes	Yes	Yes	Yes	Yes	No	Yes	No	8	3	3	FAIR
Vasconcelos 2016[76]	Yes	Yes	Yes	No	Yes	CD	Yes	Yes	Yes	Yes	Yes	No	Yes	No	10	3	1	FAIR
Vella 2017[67]	Yes	NR	NR	No	NR	Yes	Yes	Yes	Yes	Yes	Yes	Yes	Yes	No	9	2	3	FAIR
Vincent 2006[77]	Yes	NR	NR	No	NR	Yes	No	CD	Yes	Yes	Yes	No	Yes	No	6	4	4	POOR
Wong 2019[78]	Yes	Yes	Yes	NR	NR	Yes	Yes	Yes	NR	Yes	Yes	Yes	Yes	Yes	11	0	3	GOOD
Zemkova 2017[81]	Yes	NR	NR	No	NR	CD	Yes	Yes	NR	Yes	Yes	No	Yes	Yes	7	2	5	FAIR
Zhang 2017[41]	Yes	NR	NR	No	NR	Yes	Yes	Yes	Yes	Yes	Yes	No	Yes	No	8	3	3	FAIR

Criteria controlled trials: (1) Randomized study; (2) Adequate randomization method; (3) Treatment allocation concealment; (4) Blinding treatment assignment; (5) Blinding outcome assessors; (6) Similar baseline characteristics; (7) Drop-out rate <20%; (8) Differential drop-out rate between groups <15%; (9) High adherence; (10) Similar background treatments; (11) Valid and reliable outcome measures; (12) Sample size justification; (13) Pre-specified outcomes/subgroups; (14) All randomized participants analysed (ITT analysis); NR, Not Reported; NA, Not Applicable; CD, Cannot Determine.

Table S3. Characteristics of the randomised controlled trials included in the meta-analysis on the effect of aerobic exercise training on VO_{2max}.

Reference	Population	Number of participants	Sex **(%F)**	Age (y)	Intervention (aerobic training)	Comparison (no exercise)	Intervention duration	Supervision
Ahmadizad 2007[21]	Obese, sedentary mean BMI 28	16	0	Mean ~ 41	Running, 3x pw 20-30 min 75-85%HR_{max}	No exercise	12 weeks	yes
Blond 2019[10]	Overweight and obese (BMI 25-35), inactive	130	52	20-45	5x pw<br>1. Active commuting (bicycle)<br>2. Moderate activity<br>3. Vigorous activity (walking, running, rowing, cross trainer or stationary cycling)<br>F 320 kcal/d, M 420 kcal/d	No exercise	6 months	no
Brooker 2019[22]	Overweight and obese	20	50	18-60	Treadmill walking or running and other types of aerobic exercise, 250 min pw moderate-to-vigorous intensity<br>1. morning (6-9 AM), 2. evening (4-7 PM)	No exercise	12 weeks	partly
Cao 2019[23]	BMI >25, sedentary past 2 years	30	100	60-69	Walking/jogging 3x 60 min pw at maximal fat oxidation intensity	No exercise	12 weeks	yes

Chin 2020[11]	BMI > 23, BF > 20, < 3 h pw physical activity	43	0	18-30	Running, 3x pw 30 min 60% HRR	No exercise	8 weeks	yes
Church 2007[24]	BMI 25-43, postmenopausal, sedentary (not exercising > 20 minutes on ≥ 3 d/wk, and taking < 8000 steps/d assessed over the course of 1 week)	464	100	45-74	Treadmill or cycle ergometer, 3-4x pw 50% VO_{2peak}; 1. 4 kcal/kg pw 2. 8 kcal/kg pw 3. 12 kcal/kg pw	No exercise	6 months	yes
Donnelly 2013[25]	BMI 25-40, planned PA ≤ 500 kcal/wk	141	55	18-30	Treadmill walking/jogging, 5x pw 400 kcal/session, 70-80% HRmax	No exercise	10 months	yes
Duscha 2005[26]	BMI 25-35, sedentary, dyslipidemia	282	?	40-65	1. walking 19 km/wk at 40 to 55% VO_{2peak}, 2. jogging 19 km/wk at 65 to 80% VO_{2peak}, 3. Jogging 32 km/wk at 65 to 80% VO_{2peak}	No exercise	7-9 months	yes

Emerenziani 2014[27]	Obese, elderly, with T2DM, mean BMI 35, sedentary	30	?	Mean 67	Treadmill or cycle ergometer, 2x pw 50 min at VT	No exercise	3 months	yes
Gerosa-Neto 2019[28]	BMI ≥ 30, ≤ 2x pw exercise, VO_{2max} < 47 ml/min.kg	36	0	18-35	Treadmill running, 3x pw, intensity and duration unclear	No exercise	6 weeks	yes
Ho 2012[29]	BMI > 25 or WC >80/90, < 1h pw moderate intensity exercise	46	84	40-66	5x pw 30 min 60%HRR	No exercise	12 weeks	partly
Irving 2008[30]	WC≥80 plus 2 or more MetS criteria	37	100	Mean 51	Walking/running<br>1. low intensity: 3 to 5x pw, 300 to 400 kcal/session, RPE 10-12<br>2. high intensity: 3 to 4x pw, 300 to 400 kcal/session, RPE 15-17	No exercise	16 weeks	yes
Jurio-Iriarte 2018[31]	BMI ≥ 25, inactive, stage 1 or 2 hypertension	48	25	Mean 56	Treadmill or cycle ergometer; 2x pw 45 min 50-75% HRR	No exercise	1. 8 weeks<br>2. 12 weeks<br>3. 16 weeks	yes

Keating 2015[32]	BMI > 25, <3x pw exercise	48	65	29-59	Cycle ergometer and walking, 3x pw<br>1. high intensity/low volume: 30 to 45 min at 70% VO_{2max}<br>2. low intensity/high volume: 45 to 60 min at 50% VO_{2max}<br>3. low intensity/low volume: 30 to 45 min at 50% VO_{2max}	No exercise	8 weeks	partly
Kim 2016[33]	Obese, no exercise	29	40	19-35	Treadmill or cycle ergometer and treadmill mountain climber, 5x pw, 60 min 65-80% HR_{max}	No exercise	8 weeks	yes
Kirk 2003[34]	BMI 27-32, sedentary (< 500 kcal pw PA)	131	1. 100<br>2. 0	19-30	Treadmill walking, from 3x pw 20 min 60%HRR to<br>5x pw 45 min 75%HRR at 6 months	No exercise	16 months	yes
Moghadasi 2011[35]	Overweight or obese (mean BMI 32), iactive	16	0	middle -aged	Treadmill, 4x pw 45 min at 75-80% VO_{2max}	No exercise	12 weeks	NR
Nader 2016[36]	BMI ≥ 25	20	100	Mean 22	Running, 3x pw up to 3.2 km 70-75% HR_{max}	No exercise	12 weeks	NR
Ramos 2019[37]	BMI ≥ 28, hypertensive	19	100	≥ 60	Jogging, 3x pw 50 min at 60% HR_{max}	No exercise	12 weeks	NR
Rayes 2019[38]	BMI > 25, inactive	47	79	30-66	Treadmill running, 3x pw 60 min at VT	No exercise	8 weeks	yes

Reichkendler 2012[39]	BMI 25-30, sedentary, VO_{2max} < 45 ml/min.kg	61	0	20-40	Running, bicycling, elliptical training, or rowing 3x pw >70%, 4x pw 50-70%VO_{2max}<br>1. moderate dose (300 kcal/d)<br>2. high dose (600 kcal/d)	No exercise	11 weeks	no
Schroeder 2019[14]	BMI 25-40, sedentary, elevated blood pressure or hypertension	34	61	45-74	Treadmill or cycle ergometer, 3x pw 60 min, 40 up to 70% (max 80%) HRR	No exercise	8 weeks	yes
Utter 1998[40]	BMI 25-65	43	100	25-75	5x pw 45 min walking 60-80% HR_{max}	No exercise	12 weeks	yes
Zhang 2015[41]	BMI ≥ 25, BF ≥ 30, PA class 2x pw	31	100	18-22	cycle ergometer, 3-4x pw 200-300 kJ/session* at 60% VO_{2max}	No exercise	12 weeks	yes

HR_{max} = maximal heart rate; HRR = heart rate reserve; VT = ventilatory threshold; BMI is body mass index (kg/m^2); BF = body fat (%); WC = waist circumference (cm); T2DM = type 2 diabetes mellitus; MetS = metabolic syndrome; RPE = rating of perceived exertion; F = female; pw = per week; PA = physical activity; NR = not reported; * authors use kJ/session, probably is kcal/session.

Table S4. Characteristics of the randomised controlled trials included in the meta-analysis on the effect of resistance exercise training on VO_{2max}.

Reference	Population	Number of participants	Sex	Age (y)	Intervention (resistance training)	Comparison (no or sham exercise)	Intervention duration	Supervision
Ahmadizad 2007[21]	Obese, sedentary, mean BMI 28	16	0	Mean 40	3x pw 50-60 min circuit weight training, 11 exercises, 4 sets, max 12 repetitions at 50-60% 1RM	No exercise	12 weeks	yes
Batrakoulis 2018[42]	BMI 25-35, inactive	40	100	30-45	3x pw, up to 41 min circuit training 10-12 exercises, up to 3 sets, as many repetitions as possible (20-40 s)	No exercise	40 weeks	yes
Ho 2012[29]	BMI > 25 or WC >80/90, < 1h pw moderate intensity exercise	47	84	40-66	5x pw, 5 exercises 4 sets, 8-12 repetitions at 10RM	No exercise	12 weeks	no
Keating 2017[43]	BMI ≥ 25, inactive	29	86	29-59	3x pw, 10 exercises, 3 sets, 8-12 repetitions, 80-85% 1RM	Sham exercise	8 weeks	yes
Kim 2016[33]	BMI≥23 or BF ≥25(M) or 30(F)	29	40	19-35	5x pw, 5-6 exercises, 3 sets, 65-80% 1RM	No exercise	8 weeks	yes

Schroeder 2019[14]	BMI 25-40, sedentary	34	61	45-74	3x pw 60 min, 12 exercises, up to 3 sets, 10-14 maximal repetitions	No exercise	8 weeks

BMI = body mass index (kg/m^2); BF = body fat (%); T2DM = type 2 diabetes mellitus; F = female; pw = per week; 1RM = 1 repetition maximum; 10RM = 10 repetition maximum

Table S5. Characteristics of the randomised controlled trials included in the meta-analysis on the effect of combined aerobic and resistance exercise training on VO_{2max}.

Reference	Population	Number of participants	Sex (%F)	Age (y)	Intervention (combined aerobic and resistance exercise)	Comparison (no or sham exercise)	Intervention duration
Banitalebi 2019[44]	Overweight with T2DM	35	100	30-65	3x pw treadmill or cycle ergometer exercise up to 30 min/session at 70%HR_{max}<br>2-3 sets of 10-12 repetitions up to 10-RM, 5 muscle groups	No exercise	10 weeks
Bonfante 2017[47]	Overweight/ obese, no regular exercise	54	0	middle-aged	3x pw 60 min, 3 sets 6-10 max repetitions plus walking/running at 55-85% VO_{2peak}	No exercise	24 weeks
Hara 2005[45]	BMI > 25, no regular exercise	14	0	Mean 19	3x pw treadmill or cycle ergometer exercise >30 min per session at ~50% VO_{2max} plus 2-3x pw 50-60 min 7 exercises, 3 sets 10 repetitions at 80% 1RM	No exercise	5 months
Ho 2012[29]	BMI > 25 or WC >80/90, < 1h pw moderate intensity exercise	46	84	40-66	5x pw, 15 min treadmill exercise at 60% HRR and 5 exercises, 2 sets, 8-12 repetitions at 10-RM	No exercise	12 weeks

Irwin 2003[12]	BMI ≥ 25 (or ≥ 24 and BF>33%) sedentary	173	100	50-75	5x pw; at least 1x pw treadmill or cycle ergometer exercise up to 45 min at 60-75% HR_{max} plus<br>5 exercises, 2 sets of 10 repetitions<br>Other sessions at home (walking, aerobics, and bicycling)	Stretching	12 months
Park 2015[46]	Abdominal obesity, post-menopausal, no regular exercise	20	100	Post-menopausal	3x pw, running 40min up to 56-75%HRR and 3 sets 10-12 repetitions, 10 exercises up to 70% 1RM	No exercise	12 weeks
Schroeder 2019[14]	BMI 25-40, sedentary, elevated blood pressure or hypertension	35	61	45-74	3x pw 30 min treadmill or cycle ergometer exercise at up to 70% HRR plus 8 exercises, 2 sets, 10-14 maximal repetitions	No exercise	8 weeks

HRR = heart rate reserve; HR_{max} = maximal heart rate; BMI = body mass index (kg/m^2); BF = body fat (%); T2DM = type 2 diabetes mellitus; F = female; pw = per week; 1RM = 1 repetition maximum; 10RM = 10 repetition maximum

Table S6. Characteristics of the randomised controlled trials included in the meta-analysis on the effect of high-intensity interval training on VO_{2max}.

Reference	Population	Number of participants	Sex (%F)	Age y)	Intervention (aerobic training)	Comparison (no exercise)	Intervention duration	Supervision
Arad 2015[48]	BMI > 25 African-American	28	100	20-40	Cycle ergometer, 3x pw 24 min, 4 intervals, of 30-60s 75-90%HRR/180-210s 50%HRR	No exercise	14 weeks	yes
Bantitalebi 2019[44]	BMI 25-48, with T2DM, <20 min structured exercise pw	35	100	30-65	Cycle ergometer, 3x pw 4 intervals of 30 s all-out/2 min 50W	No exercise	10 weeks	yes
Chin 2020[11]	BMI > 23, BF > 20, < 3 h pw physical activity	87	0	18-30	30-m shuttle runs, 12 bouts of 1 min 90%HRR/1 min 70%HRR<br>1. 3x pw<br>2. 2x pw<br>3. 1x pw	No exercise	8 weeks	yes
Gerosa-Neto 2019[28]	BMI ≥ 30, ≤ 2x pw exercise, VO_{2max} < 47 ml/min.kg	36	0	18-35	Cycle ergometer, 3x pw, intensity and duration unclear	No exercise	6 weeks	yes
Jabbour 2015[49]	Obese, inactive (< 1 h pw)	24	46	young	Cycle ergometer, 3x pw 6 repetitions, 6s all-out /2 min passive recovery	No exercise	6 weeks	yes

Jurio-Iriarte 2018[31]	BMI ≥ 25, inactive, stage 1 or 2 hypertension	47	25	Mean 56	2x pw 45 min:<br>Treadmill 4 repetitions<br>4 min 76-95%HRR/3 min 50-75%HRR, or cycle ergometer 18 repetitions 30s high intensity/60s moderate intensity	No exercise	1. 8 weeks<br>2. 12 weeks<br>3. 16 weeks	yes
Smith-Ryan 2016[50]	BMI > 25, sedentary	32	100	18-55	Cycle ergometer, 3x pw<br>1. 10 repetitions 1 min 90% VO_{2peak}/1 min rest<br>2. 5 repetitions 2 min 80-100% VO_{2peak}/1 min rest	No exercise	3 weeks	yes
Tong 2018[51]	BF > 30, attending PE class 2x pw but no other exercise training	54	100	18-23	3-4 x pw<br>1. SIT: cycle ergometer, 80 repetitions, 6s resistance (start 1 kp, gradually increasing if 80 repetitions were sustained without undue fatigue) /9s passive recovery<br>2. prolonged HIIT: cycle ergometer, 4 min 90% VO_{2max}/3 min passive recovery up to 400 kJ/session	No exercise	12 weeks	yes
Trilk 2010[52]	BMI > 25, sedentary	28	100	Mean ~ 30	Cycle ergometer, 3x pw, 4-7 repetitions 30s resistance 0.05kg/kg at maximal rpm/4 min active recovery	No exercise	4 weeks	yes
Zhang 2015[41]	BMI ≥ 25, BF ≥ 30, PA class < 2x pw	31	100	18-22	Cycle ergometer, 3-4x pw 200-300 kJ/session* 4 min 90% VO_{2max}/3 min passive recovery	No exercise	12 weeks	yes

HRR = heart rate reserve; BMI = body mass index (kg/m^2); BF = body fat (%); T2DM = type 2 diabetes mellitus; F = female; pw = per week; SIT = sprint interval training; HIIT = high-intensity interval training; * authors use kJ/session, probably is kcal/session.

Table S7. Characteristics of the randomised controlled trials included in the meta-analysis comparing the effects of aerobic and resistance training on VO_{2max} in adults with overweight or obesity.

Reference	Population	Number of participants	Sex (%F)	Age (y)	Intervention (resistance training)	Comparison (aerobic training)	Intervention duration	Supervision
Ahmadizad 2007[21]	Obese, sedentary, mean BMI 28	16	0	Mean ~ 40	3x pw 50-60 min circuit weight training, 11 exercises, 4 sets, max 12 repetitions at 50-60% 1RM	Running, 3x pw 20-30 min 75-85%HRmax	12 weeks	yes
Ho 2012[29]	BMI > 25 or WC >80/90	51	84	40-66	5x pw, 5 exercises 4 sets, 8-12 repetitions at 10RM	5x pw 30 min 60%HRR	12 weeks	partly
Kim 2016[33]	Obese, no exercise	38	45	19-35	5x pw, 5-6 exercises, 3 sets, 65-80% 1RM	Treadmill or cycle ergometer and treadmill mountain climber, 5x pw, 60 min 65-80% HRmax	8 weeks	yes
Kim 2020[53]	BMI≥25 inactive (≤1x pw, ≤30 min)	38	0	30-64	3x pw 90 min 6 exercises 10-12 repetitions at 50% 1RM, 1-2 min rest in between	aerobic training 60 min outdoor jogging/running 65-85% HRmax	12 weeks	yes

Sawczyn 2015[54]	overweight premenopausal, no previous exercise training	29	100	40-49	3x pw 11 exercises, 8-12 repetitions 60-65% 1RM	Cycle or rowing ergometer, 3x pw 80 min, HR 136-156 bpm	16 weeks	yes
Schjerve 2008[13]	BMI > 30	26	80	> 20	3x pw 1 exercise, 4 series, 5 repetitions 90% 1RM plus 2 exercises, 3 series, 30 repetitions	Treadmill walking or running, 3x pw 47 min 60-70% HRmax	12 weeks	partly
Schroeder 2019[14]	BMI 25-40, sedentary, elevated blood pressure or hypertension	34	61	45-74	3x pw 60 min, 12 exercises, up to 3 sets, 10-14 maximal repetitions	Treadmill or cycle ergometer, 3x pw 60 min, 40 up to 70% (max 80%) HRR	8 weeks	yes
Slentz 2011[15]	BMI 26-35 sedentary (< 2 x pw), mild-to-moderate dyslipidemia	100	56	18-70	3x pw, 8 exercises, 3 sets 8–12 repetitions	Treadmill, elliptical trainers, cycle ergometer or combination, ~19.2 km/wk at 75% VO2peak	8 months	yes

HRR = heart rate reserve; HR_{max} = maximal heart rate; BMI = body mass index (kg/m^2); BF = body fat (%); WC = waist circumference (cm); F = female; pw = per week; 1RM = 1 repetition maximum; 10RM = 10 repetition maximum.

Table S8. Characteristics of the randomised controlled trials included in the meta-analysis comparing the effects of aerobic and combined aerobic plus resistance training on VO_{2max} in adults with overweight or obesity.

Reference	Population	Number of participants	Sex (%F)	Age (y)	Intervention (combined aerobic plus resistance training)	Comparison (aerobic training)	Intervention duration	Supervision
Ho 2012[29]	BMI > 25 or WC >80/90, < 1h pw moderate intensity exercise	50	84	40-66	5x pw 15 min aerobic at 60%HRR, 15 min Resistance: 3 exercises 2 sets of 10 repetitions, 5 exercises 1 set of 10 repetitions	5x pw 30 min 60%HRR	12 weeks	partly
Schroeder 2019[14]	BMI 25-40, sedentary, elevated blood pressure or hypertension	35	61	45-74	3x pw 60 min 30 min aerobic 40 up to 70% (max 80%) HRR, 30 min resistance 8 exercises, 2 sets 10-14 maximal repetitions	Treadmill or cycle ergometer, 3x pw 60 min, 40 up to 70% (max 80%) HRR	8 weeks	yes
Skrypnik 2015[55]	BMI ≥30, WC>80, BF≥33%	44	100	18-65	3x pw 60 min 20 min variable strength exercises plus 25 min aerobic exercise cycle ergometer at 50-80% HRmax	Cycle ergometer, 3x pw 60 min 50-80%HRmax	3 months	yes

Slentz 2011[15]	BMI 26-35 sedentary (< 2 x pw), mild-to-moderate dyslipidemia	92	56	18-70	Treadmill, elliptical trainers, cycle ergometer or combination, 3x pw~19.2 km/wk (12 miles/wk) at 75% VO_{2peak} plus 8 exercises, 3 sets 8–12 repetitions	Treadmill, elliptical trainers, cycle ergometer or combination, 3x pw ~19.2 km/wk (12 miles/wk) at 75% VO_{2peak}	8 months	yes

HRR = heart rate reserve; HR_{max} = maximal heart rate; BMI = body mass index (kg/m^2); BF = body fat (%); WC = waist circumference (cm); F = female; pw = per week.

Table S9. Characteristics of the randomised controlled trials included in the meta-analysis comparing the effects of aerobic and high-intensity interval (HIIT) training on VO_{2max} in adults with overweight or obesity.

Reference	Population	Number of participants	Sex	Age (y)	Intervention (HIIT)	Comparison (aerobic training)	Intervention duration	Supervision
Baekkerud 2016[56]	BMI ≥ 25	30	60	≥ 18	3x pw 45 min treadmill running/walking<br>1. 4 intervals 4 min 85-95% HR_{max}/3 min 70% HR_{max}<br>2. 10 intervals 1 min 90% HR_{max}	3x pw 45 min treadmill running/walking70% HR_{max} (EE equivalent with HIIT group 1)	6 weeks	yes
Cheema 2015[57]	BMI > 25, WC > 94 (M) or > 80 (F), inactive (<3x pw)	12	50	> 18	4x pw 50 min HIIT boxing (2 min/1 min rest)<br>3 sets of 5 exercises, RPE 15-17/20	4x pw 50 min brisk walking (as fast as possible)	12 weeks	partly
Chin 2020[11]	BMI≥23, BF>20, <3h pw PA	76	0	18-30	30-m shuttle runs 12 bouts 90% HRR/1 min recovery 70% HRR<br>1. 3x pw<br>2. 2x pw<br>3. 1x pw	3x pw 30 min 60% HRR	8 weeks	yes

Cocks 2016[58]	BMI≥30, sedentary (≤1h structured PA pw)	16	0	young	Cycle ergometer 3x pw 4-7 30-s intervals 200% W_{max}/120s 30W	Cycle ergometer 5x pw 40-60 min ~65% VO_{2max}	4 weeks	NR
De Strijcker 2018[59]	BMI 28-36, HbA1c< 6.5%, sedentary (< 1h pw)	16	0	42-57	Cycle ergometer 2x pw 40 min,<br>2x 10 15-s intervals 100 rpm, resistance 100-110% HR_{VT}/45s 40-60 rpm, resistance 50-55% HR_{VT}, 1x 10 min 60 rpm, 100-110% HR_{VT}	Cycle ergometer 2x pw 40 min, 60 rpm, resistance 100-110% HR_{VT}	10 weeks	yes
Fisher 2015[60]	BMI 25-35, sedentary (< 30 min structured PA pw)	28	0	17-22	Cycle ergometer 3x pw 20 min<br>4 sets 4 min 15% PP/30s 85% PP	Cycle ergometer 5x pw 45-60 min 55-65% VO_{2max}	6 weeks	yes
Gerosa-Neto 2019[28]	BMI ≥ 30, ≤ 2x pw exercise, VO_{2max} < 47 ml/min.kg	36	0	18-35	Cycle ergometer, 3x pw, intensity and duration unclear	3x pw 30 min 65% VO_{2max} (EE matched with HIIT)	6 weeks	NR
Higgins 2016[65]	BMI ≥ 25, < 2x pw < 30min PA	60	100	18-24	Cycle ergometer, 3x pw 30s all-out/4 min recovery, 5-7 repetitions	Cycle ergometer, 3x pw 20-30 min at 60-70% HRR	6 weeks	yes

Jurio-Iriarte 2018[31]	BMI ≥ 25, inactive, stage 1 or 2 hypertension	45	25	Mean 56	2x pw 45 min<br>Treadmill 4 repetitions 4 min 76-95%HRR/3 min 50-75%HRR, or cycle ergometer 18 repetitions 30s high intensity/60s moderate intensity	Treadmill or cycle ergometer; 2x pw 45 min 50-70% HRR	1. 8 weeks<br>2. 12 weeks<br>3. 16 weeks	yes
Kong 2016[61]	BMI > 23 and BF > 30%	22	100	18-30	Cycle ergometer, 4x pw 60 repetitions 8s up to 0.05xBW/12s recovery	Cycle ergometer, 4x pw 40 min 65% VO_{2max}	5 weeks	yes
Lunt 2014[62]	BMI 28-40, <2x pw 30 min	49	74	35-60	Outside walking or jogging, 3x pw<br>1. Fast walking or jogging, 4 min 85-95% HR_{max}/3 min walking, 4 repetitions<br>2. 30-s all-out walking or jogging/4 min recovery walking, 3 repetitions	Outside walking, 3x pw 33 min 65-75% HR_{max}	12 weeks	yes
Martins 2016[63]	Obese (mean BMI 33.3), sedentary	46	65	Mean 34.4	Cycle ergometer, 3x pw<br>1. 10 repetitions. 8s allout/12s 85-90% HR_{max}, 250 kcal<br>2. half of 1, 125 kcal.	Cycle ergometer, 70% HR_{max} (250 kcal)	12 weeks	yes
Robinson 2015[66]	BMI > 24, inactive (<2x 30 min pw), prediabetes	39	82	NR	5x pw, 4 up to 10 repetitions 1 min at 85-90% W_{peak}/1 min recovery 20% W_{peak}	Cycle ergometer, treadmill or outdoor walking, elliptical trainer, 5x pw at 32.5% W_{peak}	2 weeks	partly

Sawyer 2016[87]	BMI ≥ 30, inactive	22	50	18-55	Cycle ergometer, 3x pw 10 repetitions 1 min at 90-95% HR_{max}/1 min 25-50W	Cycle ergometer, 3x pw 30 min at 70-75% HR_{max}	8 weeks	NR
Schjerve 2008[13]	BMI > 30	27	80	> 20	Treadmill walking or running, 4 intervals 4 min 85-95% HR_{max}/3 min 50-60% HR_{max}	Treadmill walking or running, 3x pw 47 min 60-70% HR_{max}	12 weeks	partly
Sun 2019[64]	BMI ≥ 23, BF ≥ 30, sedentary	48	100	19-25	Cycle ergometer 3x pw<br>1. 80 repetitions 6s all-out, >100 rpm, 9s recovery, up to 5% of BM<br>2. 4 min 90% W_{max}/3 min passive recovery, matched for EE with 1.	Cycle ergometer, 3x pw 60% W_{max} matched for EE with 1.	12 weeks	yes
Vella 2017[67]	overweight or obesity, sedentary	19	58	18-44	Treadmill, cycle ergometer, elliptical trainer, 4x pw, 10 repetitions, 1 min 75-80% HRR/1 min recovery 35-40% HRR	Treadmill, cycle ergometer, elliptical trainer, 4x pw, 20 min at 55-59% HRR	8 weeks	partly
Zhang 2015[41]	BMI ≥ 25, BF ≥ 30, PA class 2x pw	32	100	18-22	Cycle ergometer, 3x pw 200-300 kJ/session, 4 min 90% VO_{2max}/3 min passive rest	Cycle ergometer, 3x pw 200-300 kJ/session, 60% VO_{2max}	12 weeks	yes

HRR = heart rate reserve; HR_{max} = maximal heart rate; BMI = body mass index (kg/m^2); BF = body fat (%); WC = waist circumference (cm); BM = body mass; F = female; pw = per week; PA = physical activity; HR_{VT} = heart rate at ventilatory threshold; HRR = heart rate reserve; PP = peak power from Wingate test; EE = energy expenditure; W_{peak} = maximal aerobic power; NR = not recorded.

Table S10. Characteristics of the randomised controlled trials included in the meta-analysis on the effects of aerobic training on muscle strength in adults with overweight or obesity.

Reference	Population	Number of participants	Sex (%F)	Age (y)	Intervention (aerobic training)	Comparison	Outcome	Intervention duration	Supervision
Cao 2019[23]	BMI >25, sedentary past 2 years	30	100	60-69	Walking/jogging 3x pw 60 min at maximal fat oxidation intensity	No exercise	Hand grip (kg)	12 weeks	yes
Chen 2017[70]	BMI ≥ 25, sarcopenic obesity	44	84	65-75	Aerobics, 2x pw 60 min	No exercise	1. hand grip (kg)<br>2. maximum back extensor strength (kg)<br>3. maximum knee extensor strength (kg)	8 weeks	yes
Kim 2016[33]	BMI ≥ 23 or BF ≥ 25 (M) or ≥ 30 (F), no regular moderate/vigorous exercise past 3 months	29	35	19-35	Treadmill, cycle ergometer or mountain climber, 5x pw 60 min at 65-80% HR_{max}	No exercise	1.Extensor right (Nm/kg)<br>2. Flexor right (Nm/kg)<br>3. Hand grip (kg/kg)	8 weeks	yes

Ramos 2019[37]	BMI ≥ 28, hypertensive	19	100	≥ 60	Jogging, 3x pw 50 min at 60% HR_{max}	No exercise	Jump height (cm)	12 weeks	yes
Sarsan 2006[71]	BMI ≥ 30, no current exercise training	50	100	20-60	Cycle ergometer, 3x pw 12-15 min up to 5x pw 30-45 min at 50-85% HRR	No exercise	1. 1RM quadriceps<br>2. 1RM biceps	13 weeks	yes
Schroeder 2019[14]	BMI 25-40, sedentary, elevated blood pressure or hypertension	34	F and M	45-74	Treadmill or cycle ergometer, 3x pw 60 min, 40 up to 70% (max 80%) HRR	No exercise	1. lower body 1RM (kg)<br>2. upper body 1RM (kg)	8 weeks	yes

HR_{max} = maximal heart rate; HRR = heart rate reserve; BMI = body mass index (kg/m^2); BF = body fat (%); M = male, F = female; pw = per week; PA = physical activity.

Table S11. Characteristics of the randomised controlled trials included in the meta-analysis on the effects of resistance training on muscle strength in adults with overweight or obesity.

Reference	Population	Number of partici-pants	Sex	Age (y)	Intervention (resistance training)	Comparison (no exercise)	Outcome	Intervention duration	Supervision
Batrakoulis 2018[42]	BMI 25-35, inactive	40	100	30-45	3x pw, up to 41 min circuit training 10-12 exercises, up to 3 sets, as many repetitions as possible (20-40 s)	No exercise	Leg 1RM (kg)	40 weeks	yes
Chen 2017[70]	BMI ≥ 25, sarcopenic obesity	46	83	65-75	2x pw 10 exercises, 3 sets of 10-12 repetitions at 60-70% 1RM	No exercise	1. hand grip (kg)<br>2. back extensor (kg)<br>3. knee extensor (kg)	8 weeks	yes
Fritz 2018[72]	BMI ≥ 25, sedentary (< 60 min pw PA)	75	100	60-75	2x pw, 6 exercises, 3-4 sets, 10 repetitions, RPE 7-9<br>1. normal elastic band<br>2. elastic tube with handles	No exercise	1. upright rowing (kg)<br>2. squat (kg)<br>3. trunk extension (kg)	8 weeks	yes
Kim 2016[33]	BMI ≥ 23 or BF ≥ 25 (M) or ≥ 30 (F), no regular moderate/vi gorous	29	40	19-35	5x pw, 5-6 exercises, 3sets, 10-12 repetitions at 65-80% 1RM	No exercise	1.Extensor right (Nm/kg)<br>2. Flexor right (Nm/kg)<br>3. Hand grip (kg/kg)	8 weeks	yes

	exercise past 3 months								
Liao 2018[73]	Obesity and (pre)sarcopenia	56	100	60-80	3x pw, elastic band exercises, 3 sets of 10 repetitions, gentle concentric and eccentric contractions through full range of motion	No exercise	1. hand grip (kg/kg)<br>2. leg (N/kg)	12 weeks	yes
Plotnikoff 2010[74]	BMI > 30, with T2DM, sedentary	48	67	?	3x pw, 8 exercises, 2-3 sets, up to 8-10 repetitions at 85% 1RM	No exercise	1. Bench press 1RM (kg)<br>2. Leg press 1RM (kg)<br>3. Upright row 1RM (kg)	16 weeks	yes
Rustaden 2017[75]	BMI ≥ 25, < 2x pw PA	143	100	18-65	3x pw, 9 exercises, 50-100 repetitions, 45-60 min<br>1. Body pump<br>2. Resistance supervised<br>3. Resistance unsupervised	No exercise	1. Squat 1RM (kg)<br>2. Bench press 1RM (kg)	12 weeks	yes

Sarsan 2006[71]	BMI ≥ 30, no current exercise training	50	100	20-60	3x pw 6 exercises, from 1 set of 10 repetitions at 40-60% 1RM up to 3 sets of 10 repetitions at 75-80% 1RM	No exercise	1. 1RM quadriceps<br>2. 1RM biceps	13 weeks	yes
Schroeder 2019[14]	BMI 25-40, sedentary, elevated blood pressure or hypertension	34	F and M	45-74	3x pw 60 min, 12 exercises, up to 3 sets 10-14 maximal repetitions	No exercise	1. lower body 1RM (kg)<br>2. upper body 1RM (kg)	8 weeks	yes
Vasconcelos 2016[76]	BMI ≥ 30, sarcopenic obesity (handgrip strength ≤ 21 kg)	31	F	65-80	2x pw 60 min, concentric and eccentric exercises as fast as possible (isokinetic)	No exercise	Knee extensor (J/kg)	10 weeks	yes
Vincent 2006[77]	BMI > 25	40(?)	F and M	60-83	3x pw, 1 set of 13 exercises, 8-13 repetitions at 50-80% 1RM	No exercise	1. Upper body (Nm)<br>2. Lower body (Nm)	24 weeks	yes
Wong 2009[78]	BMI > 30 and ≤ 40, sedentary (< 1h/wk), postmeno-pausal	20	F	50-65	3x pw, 4 exercises, 2-3 sets, 18-22 repetitions until fatigue	No exercise	Leg press 8RM (kg)	12 weeks	yes

BMI = body mass index (kg/m^2); BF = body fat (%); M = male, F = female; pw = per week; PA = physical activity, T2DM = type 2 diabetes mellitus.

Table S12. Characteristics of the randomised controlled trials included in the meta-analysis on the effects of combined aerobic plus resistance training on muscle strength in adults with overweight or obesity.

Reference	Population	Number of partici-pants	Sex (%F)	Age (y)	Intervention (combined aerobic plus resistance training)	Comparison (no exercise)	Outcome	Intervention duration	Supervision
Bonfante 2017[47]	overweight/obese, no regular exercise	54	0	middle-aged	3x pw 60 min, 3 sets of 6-10 max repetitions plus walking/running at 55-85% VO_{2peak}	No exercise	1. 1RM leg press (kg)<br>2. 1RM bench press (kg)<br>3. 1 RM arm curl (kg)	24 weeks	yes
Chen 2017[70]	BMI ≥ 25, sarcopenic obesity	47	83	65-75	1x pw 10 exercises, 3 sets of 10-12 repetitions at 60-70% 1RM plus 1x pw 60 min aerobics	No exercise	1. hand grip (kg)<br>2. maximum back extensor strength (kg)<br>3. maximum knee extensor strength (kg)	8 weeks	yes
Kang 2012[79]	Obese, no exercise	12	100	21-23	3x pw 40-65 min, circuit weight training plus jogging at 50-70% HRR	No exercise	1. back (kg)<br>2. hand grip (kg)	12 weeks	NR

Park 2015[46]	Abdominal obesity, postmenopausal, no regular exercise	20	100	Middle-aged	3x pw 30-40 min treadmill, up to 75% HRR plus 30 minutes of resistance exercises, 3 sets 10-12 repetitions up to 70% 1RM	No exercise	1. hand grip (kg)<br>2. Back (kg)	12 weeks	NR
Park 2017[80]	BMI ≥ 25, sarcopenia	50	100	≥ 65	5x pw 50-80 min, elastic band 2-3 sets up to 12-15 repetitions (3x pw) plus walking 30-50 min at RPE 13-17	No exercise	Hand grip right (kg)	24 weeks	yes
Schroeder 2019[14]	BMI 25-40, sedentary, elevated blood pressure or hypertension	35	61	45-74	3x pw 30 min 12 exercises, up to 3 sets 10-14 maximal repetitions plus 3x pw 30 min treadmill or cycle ergometer up to 70% HRR	No exercise	1. lower body 1RM (kg)<br>2. upper body 1RM (kg)	8 weeks	yes

BMI = body mass index (kg/m^2); M = male, F = female; pw = per week; NR = not recorded; HRR = heart rate reserve; RPE = rating of perceived exertion; 1RM = 1 repetition maximum.

Table S13. Characteristics of the randomised controlled trials included in the meta-analysis comparing the effects of resistance training and aerobic training on muscle strength in adults with overweight or obesity.

Reference	Population	Number of partici-pants	Sex (% F)	Age (y)	Intervention (resistance training)	Comparison (aerobic training)	Outcome	Intervention duration	Supervision
Chen 2017[70]	BMI ≥ 25, sarcopenic obesity	46	83	65-75	2x pw 10 exercises, 3 sets of 10-12 repetitions at 60-70% 1RM	2x pw 60 min aerobics	1. hand grip (kg)<br>2. maximum back extensor strength (kg)<br>3. maximum knee extensor strength (kg)	8 weeks	yes
Kim 2016[33]	BMI ≥ 23 or BF ≥ 25 (M) or ≥ 30 (F), no regular moderate/vigo-rous exercise past 3 months	38	45	19-35	5x pw, 5-6 exercises, 3sets, 10-12 repetitions at 65-80% 1RM	Treadmill or cycle ergometer and treadmill mountain climber, 5x pw, 60 min 65-80% HRmax	1.Extensor right (Nm/kg)<br>2. Flexor right (Nm/kg)<br>3. Hand grip (kg/kg)	8 weeks	yes
Kim 2020[53]	BMI ≥ 25, inactive (≤ 1x pw, ≤ 30 min)	38	0	30-64	3x pw, 90 min, 7 exercises, 3 sets, 10-12 repetitions at 50% 1RM	Outdoor jogging/running, 3x pw 60 min upto 65-85% HR_{max}	Knee extensor (Nm/kg)	12 weeks	yes
Sarsan 2006[71]	BMI ≥ 30, no current exercise training	52	100	20-60	3x pw 6 exercises, from 1 set of 10 repetitions at 40-60% 1RM up to 3 sets of	Cycle ergometer, from 3x pw 12-15 min to 5x pw 30-	1. 1RM quadriceps<br>2. 1RM biceps	13 weeks	yes

					10 repetitions at 75-80% 1RM	45 min at 50-85% HRR			
Schjerve 2008[13]	BMI > 30	26	80	> 20	3x pw 1 exercise, 4 series, 5 repetitions at 90% 1RM and 2 exercises, 3 series, 30 repetitions	Treadmill walking or running, 3x pw 47 min 60-70% HR_{max}	Leg 1RM (kg)	12 weeks	partly
Schroeder 2019[14]	BMI 25-40, sedentary, elevated blood pressure or hypertension	34	61	45-74	3x pw 60 min, 12 exercises, up to 3 sets 10-14 maximal repetitions	Treadmill or cycle ergometer, 3x pw 60 min, 40 up to 70% (max 80%) HRR	1. lower body 1RM (kg)<br>2. upper body 1RM (kg)	8 weeks	yes
Zemkova 2017[81]	Overweight/ obese	17	0	Mean 38	2-3x pw, 4-5 exercises, 2-5 sets, 8-12 repetitions, 57-82% 1RM	Aerobic dancing, running or spinning, 3x pw 60 min at 70-85% HR_{max}	1. leg press (N)<br>2. squat jump (W/kg)	3 months	yes

BMI = body mass index (kg/m^2); BF = body fat (%); M = male, F = female; pw = per week; HR_{max} = maximal heart rate; HRR = heart rate reserve; 1RM = 1 repetition maximum.

References

1 Sui, X. *et al.* Cardiorespiratory fitness and adiposity as mortality predictors in older adults. *JAMA* **298**, 2507-2516, doi:10.1001/jama.298.21.2507 (2007).

2 Tomlinson, D. J., Erskine, R. M., Morse, C. I., Winwood, K. & Onambele-Pearson, G. The impact of obesity on skeletal muscle strength and structure through adolescence to old age. *Biogerontology* **17**, 467-483, doi:10.1007/s10522-015-9626-4 (2016).

3 Wang, C. Y. *et al.* Cardiorespiratory fitness levels among US adults 20-49 years of age: findings from the 1999-2004 National Health and Nutrition Examination Survey. *Am J Epidemiol* **171**, 426-435, doi:10.1093/aje/kwp412 (2010).

4 Miller, C. T. *et al.* The effects of exercise training in addition to energy restriction on functional capacities and body composition in obese adults during weight loss: a systematic review. *PLoS One* **8**, e81692, doi:10.1371/journal.pone.0081692 (2013).

5 Baker, A., Sirois-Leclerc, H. & Tulloch, H. The Impact of Long-Term Physical Activity Interventions for Overweight/Obese Postmenopausal Women on Adiposity Indicators, Physical Capacity, and Mental Health Outcomes: A Systematic Review. *J Obes* **2016**, 6169890, doi:10.1155/2016/6169890 (2016).

6 Batacan, R. B., Jr., Duncan, M. J., Dalbo, V. J., Tucker, P. S. & Fenning, A. S. Effects of high-intensity interval training on cardiometabolic health: a systematic review and meta-analysis of intervention studies. *Br J Sports Med* **51**, 494-503, doi:10.1136/bjsports-2015-095841 (2017).

7 Hita-Contreras, F. *et al.* Effect of exercise alone or combined with dietary supplements on anthropometric and physical performance measures in community-dwelling elderly people with sarcopenic obesity: A meta-analysis of randomized controlled trials. *Maturitas* **116**, 24-35, doi:10.1016/j.maturitas.2018.07.007 (2018).

8 Turk, Y. *et al.* High intensity training in obesity: a Meta-analysis. *Obes Sci Pract* **3**, 258-271, doi:10.1002/osp4.109 (2017).

9 Su, L. *et al.* Effects of HIIT and MICT on cardiovascular risk factors in adults with overweight and/or obesity: A meta-analysis. *PLoS One* **14**, e0210644, doi:10.1371/journal.pone.0210644 (2019).

10 Blond, M. B. *et al.* How does 6 months of active bike commuting or leisure-time exercise affect insulin sensitivity, cardiorespiratory fitness and intra-abdominal fat? A randomised controlled trial in individuals with overweight and obesity. *Br J Sports Med* **53**, 1183-1192, doi:10.1136/bjsports-2018-100036 (2019).

11 Chin, E. C. *et al.* Low-Frequency HIIT Improves Body Composition and Aerobic Capacity in Overweight Men. *Med Sci Sports Exerc* **52**, 56-66, doi:10.1249/MSS.0000000000002097 (2020).

12 Irwin, M. L. *et al.* Effect of exercise on total and intra-abdominal body fat in postmenopausal women: a randomized controlled trial. *JAMA* **289**, 323-330, doi:10.1001/jama.289.3.323 (2003).

13 Schjerve, I. E. *et al.* Both aerobic endurance and strength training programmes improve cardiovascular health in obese adults. *Clin Sci (Lond)* **115**, 283-293, doi:10.1042/CS20070332 (2008).
14 Schroeder, E. C., Franke, W. D., Sharp, R. L. & Lee, D. C. Comparative effectiveness of aerobic, resistance, and combined training on cardiovascular disease risk factors: A randomized controlled trial. *PLoS One* **14**, e0210292, doi:10.1371/journal.pone.0210292 (2019).
15 Slentz, C. A. *et al.* Effects of aerobic vs. resistance training on visceral and liver fat stores, liver enzymes, and insulin resistance by HOMA in overweight adults from STRRIDE AT/RT. *Am J Physiol Endocrinol Metab* **301**, E1033-1039, doi:10.1152/ajpendo.00291.2011 (2011).
16 Review Manager (RevMan). Version 5.3 (The Nordic Cochrane Centre, The Cochrane Collaboration, Copenhagen, 2014).
17 Cohen, J. *Statistical power analysis for the behavioral sciences*. 2nd edn, (Lawrence Erlbaum Associates, 1988).
18 Higgins, J. P. T. & Green, S. (2011).
19 Patsopoulos, N. A., Evangelou, E. & Ioannidis, J. P. Sensitivity of between-study heterogeneity in meta-analysis: proposed metrics and empirical evaluation. *Int J Epidemiol* **37**, 1148-1157, doi:10.1093/ije/dyn065 (2008).
20 Jensen, M. D. *et al.* 2013 AHA/ACC/TOS guideline for the management of overweight and obesity in adults: a report of the American College of Cardiology/American Heart Association Task Force on Practice Guidelines and The Obesity Society. *Circulation* **129**, S102-138, doi:10.1161/01.cir.0000437739.71477.ee (2014).
21 Ahmadizad, S., Haghighi, A. H. & Hamedinia, M. R. Effects of resistance versus endurance training on serum adiponectin and insulin resistance index. *Eur J Endocrinol* **157**, 625-631, doi:10.1530/EJE-07-0223 (2007).
22 Brooker, P. G., Gomersall, S. R., King, N. A. & Leveritt, M. D. The feasibility and acceptability of morning versus evening exercise for overweight and obese adults: A randomized controlled trial. *Contemp Clin Trials Commun* **14**, 100320, doi:10.1016/j.conctc.2019.100320 (2019).
23 Cao, L., Jiang, Y., Li, Q., Wang, J. & Tan, S. Exercise Training at Maximal Fat Oxidation Intensity for Overweight or Obese Older Women: A Randomized Study. *J Sports Sci Med* **18**, 413-418 (2019).
24 Church, T. S., Earnest, C. P., Skinner, J. S. & Blair, S. N. Effects of different doses of physical activity on cardiorespiratory fitness among sedentary, overweight or obese postmenopausal women with elevated blood pressure: a randomized controlled trial. *JAMA* **297**, 2081-2091, doi:10.1001/jama.297.19.2081 (2007).
25 Donnelly, J. E. *et al.* Aerobic exercise alone results in clinically significant weight loss for men and women: midwest exercise trial 2. *Obesity (Silver Spring)* **21**, E219-228, doi:10.1002/oby.20145 (2013).
26 Duscha, B. D. *et al.* Effects of exercise training amount and intensity on peak oxygen consumption in middle-age men and women at risk for cardiovascular disease. *Chest* **128**, 2788-2793, doi:10.1378/chest.128.4.2788 (2005).
27 Emerenziani, G. P. *et al.* Effects of Aerobic Exercise Based upon Heart Rate at Aerobic Threshold in Obese Elderly Subjects with Type 2 Diabetes. *Int J Endocrinol* **2015**, 695297, doi:10.1155/2015/695297 (2015).

28 Gerosa-Neto, J. *et al.* High- or moderate-intensity training promotes change in cardiorespiratory fitness, but not visceral fat, in obese men: A randomised trial of equal energy expenditure exercise. *Respir Physiol Neurobiol* **266**, 150-155, doi:10.1016/j.resp.2019.05.009 (2019).

29 Ho, S. S., Dhaliwal, S. S., Hills, A. P. & Pal, S. The effect of 12 weeks of aerobic, resistance or combination exercise training on cardiovascular risk factors in the overweight and obese in a randomized trial. *BMC Public Health* **12**, 704, doi:10.1186/1471-2458-12-704 (2012).

30 Irving, B. A. *et al.* Effect of exercise training intensity on abdominal visceral fat and body composition. *Med Sci Sports Exerc* **40**, 1863-1872, doi:10.1249/MSS.0b013e3181801d40 (2008).

31 Jurio-Iriarte, B. & Maldonado-Martin, S. Effects of Different Exercise Training Programs on Cardiorespiratory Fitness in Overweight/Obese Adults With Hypertension: A Pilot Study. *Health Promot Pract* **20**, 390-400, doi:10.1177/1524839918774310 (2019).

32 Keating, S. E. *et al.* Effect of aerobic exercise training dose on liver fat and visceral adiposity. *J Hepatol* **63**, 174-182, doi:10.1016/j.jhep.2015.02.022 (2015).

33 Kim, H. J. *et al.* Effect of aerobic training and resistance training on circulating irisin level and their association with change of body composition in overweight/obese adults: a pilot study. *Physiol Res* **65**, 271-279, doi:10.33549/physiolres.932997 (2016).

34 Kirk, E. P., Jacobsen, D. J., Gibson, C., Hill, J. O. & Donnelly, J. E. Time course for changes in aerobic capacity and body composition in overweight men and women in response to long-term exercise: the Midwest Exercise Trial (MET). *Int J Obes Relat Metab Disord* **27**, 912-919, doi:10.1038/sj.ijo.0802317 (2003).

35 Moghadasi, M. *et al.* High-intensity endurance training improves adiponectin mRNA and plasma concentrations. *Eur J Appl Physiol* **112**, 1207-1214, doi:10.1007/s00421-011-2073-2 (2012).

36 Nader, S., Shahram, S. & Elham, Y. The effect of endurance training on adiponectin and insulin resistance in overweight female university students. *Acta Medica Mediterranea* **32**, 1007-1010 (2016).

37 Ramos, R. M. *et al.* Impact of Moderate Aerobic Training on Physical Capacities of Hypertensive Obese Elderly. *Gerontol Geriatr Med* **5**, 2333721419859691, doi:10.1177/2333721419859691 (2019).

38 Rayes, A. B. R. *et al.* The effects of Pilates vs. aerobic training on cardiorespiratory fitness, isokinetic muscular strength, body composition, and functional tasks outcomes for individuals who are overweight/obese: a clinical trial. *PeerJ* **7**, e6022, doi:10.7717/peerj.6022 (2019).

39 Reichkendler, M. H. *et al.* Only minor additional metabolic health benefits of high as opposed to moderate dose physical exercise in young, moderately overweight men. *Obesity (Silver Spring)* **22**, 1220-1232, doi:10.1002/oby.20226 (2014).

40 Utter, A. C., Nieman, D. C., Shannonhouse, E. M., Butterworth, D. E. & Nieman, C. N. Influence of diet and/or exercise on body composition and cardiorespiratory fitness in obese women. *Int J Sport Nutr* **8**, 213-222, doi:10.1123/ijsn.8.3.213 (1998).

41 Zhang, H. *et al.* Comparable Effects of High-Intensity Interval Training and Prolonged Continuous Exercise Training on Abdominal Visceral Fat Reduction in Obese Young Women. *J Diabetes Res* **2017**, 5071740, doi:10.1155/2017/5071740 (2017).

42 Batrakoulis, A. *et al.* High intensity, circuit-type integrated neuromuscular training alters energy balance and reduces body mass and fat in obese women: A 10-month training-detraining randomized controlled trial. *PLoS One* **13**, e0202390, doi:10.1371/journal.pone.0202390 (2018).
43 Keating, S. E. *et al.* Effect of resistance training on liver fat and visceral adiposity in adults with obesity: A randomized controlled trial. *Hepatol Res* **47**, 622-631, doi:10.1111/hepr.12781 (2017).
44 Banitalebi, E., Kazemi, A., Faramarzi, M., Nasiri, S. & Haghighi, M. M. Effects of sprint interval or combined aerobic and resistance training on myokines in overweight women with type 2 diabetes: A randomized controlled trial. *Life Sci* **217**, 101-109, doi:10.1016/j.lfs.2018.11.062 (2019).
45 Hara, T. *et al.* Body composition is related to increase in plasma adiponectin levels rather than training in young obese men. *Eur J Appl Physiol* **94**, 520-526, doi:10.1007/s00421-005-1374-8 (2005).
46 Park, S. M., Kwak, Y. S. & Ji, J. G. The Effects of Combined Exercise on Health-Related Fitness, Endotoxin, and Immune Function of Postmenopausal Women with Abdominal Obesity. *J Immunol Res* **2015**, 830567, doi:10.1155/2015/830567 (2015).
47 Bonfante, I. L. *et al.* Combined training, FNDC5/irisin levels and metabolic markers in obese men: A randomised controlled trial. *Eur J Sport Sci* **17**, 629-637, doi:10.1080/17461391.2017.1296025 (2017).
48 Arad, A. D. *et al.* High-intensity interval training without weight loss improves exercise but not basal or insulin-induced metabolism in overweight/obese African American women. *J Appl Physiol (1985)* **119**, 352-362, doi:10.1152/japplphysiol.00306.2015 (2015).
49 Jabbour, G., Iancu, H. D. & Paulin, A. Effects of High-Intensity Training on Anaerobic and Aerobic Contributions to Total Energy Release During Repeated Supramaximal Exercise in Obese Adults. *Sports Med Open* **1**, 36, doi:10.1186/s40798-015-0035-7 (2015).
50 Smith-Ryan, A. E., Trexler, E. T., Wingfield, H. L. & Blue, M. N. Effects of high-intensity interval training on cardiometabolic risk factors in overweight/obese women. *J Sports Sci* **34**, 2038-2046, doi:10.1080/02640414.2016.1149609 (2016).
51 Tong, T. K. *et al.* Comparing Time Efficiency of Sprint vs. High-Intensity Interval Training in Reducing Abdominal Visceral Fat in Obese Young Women: A Randomized, Controlled Trial. *Front Physiol* **9**, 1048, doi:10.3389/fphys.2018.01048 (2018).
52 Trilk, J. L., Singhal, A., Bigelman, K. A. & Cureton, K. J. Effect of sprint interval training on circulatory function during exercise in sedentary, overweight/obese women. *Eur J Appl Physiol* **111**, 1591-1597, doi:10.1007/s00421-010-1777-z (2011).
53 Kim, B. & Kim, S. Influences of Resistance versus Aerobic Exercise on Physiological and Physical Fitness Changes in Previously Inactive Men with Obesity: A Prospective, Single-Blinded Randomized Controlled Trial. *Diabetes Metab Syndr Obes* **13**, 267-276, doi:10.2147/DMSO.S231981 (2020).
54 Sawczyn, S. *et al.* Strength and aerobic training in overweight females in Gdansk, Poland. *Open Med (Wars)* **10**, 152-162, doi:10.1515/med-2015-0021 (2015).
55 Skrypnik, D. *et al.* Effects of Endurance and Endurance Strength Training on Body Composition and Physical Capacity in Women with Abdominal Obesity. *Obes Facts* **8**, 175-187, doi:10.1159/000431002 (2015).

56 Baekkerud, F. H. *et al.* Comparison of Three Popular Exercise Modalities on V O2max in Overweight and Obese. *Med Sci Sports Exerc* **48**, 491-498, doi:10.1249/MSS.0000000000000777 (2016).

57 Cheema, B. S., Davies, T. B., Stewart, M., Papalia, S. & Atlantis, E. The feasibility and effectiveness of high-intensity boxing training versus moderate-intensity brisk walking in adults with abdominal obesity: a pilot study. *BMC Sports Sci Med Rehabil* **7**, 3, doi:10.1186/2052-1847-7-3 (2015).

58 Cocks, M. *et al.* Sprint interval and moderate-intensity continuous training have equal benefits on aerobic capacity, insulin sensitivity, muscle capillarisation and endothelial eNOS/NAD(P)Hoxidase protein ratio in obese men. *J Physiol* **594**, 2307-2321, doi:10.1113/jphysiol.2014.285254 (2016).

59 De Strijcker, D. *et al.* High intensity interval training is associated with greater impact on physical fitness, insulin sensitivity and muscle mitochondrial content in males with overweight/obesity, as opposed to continuous endurance training: a randomized controlled trial. *J Musculoskelet Neuronal Interact* **18**, 215-226 (2018).

60 Fisher, G. *et al.* High Intensity Interval- vs Moderate Intensity- Training for Improving Cardiometabolic Health in Overweight or Obese Males: A Randomized Controlled Trial. *PLoS One* **10**, e0138853, doi:10.1371/journal.pone.0138853 (2015).

61 Kong, Z., Sun, S., Liu, M. & Shi, Q. Short-Term High-Intensity Interval Training on Body Composition and Blood Glucose in Overweight and Obese Young Women. *J Diabetes Res* **2016**, 4073618, doi:10.1155/2016/4073618 (2016).

62 Lunt, H. *et al.* High intensity interval training in a real world setting: a randomized controlled feasibility study in overweight inactive adults, measuring change in maximal oxygen uptake. *PLoS One* **9**, e83256, doi:10.1371/journal.pone.0083256 (2014).

63 Martins, C. *et al.* High-Intensity Interval Training and Isocaloric Moderate-Intensity Continuous Training Result in Similar Improvements in Body Composition and Fitness in Obese Individuals. *Int J Sport Nutr Exerc Metab* **26**, 197-204, doi:10.1123/ijsnem.2015-0078 (2016).

64 Sun, S. *et al.* Twelve weeks of low volume sprint interval training improves cardio-metabolic health outcomes in overweight females. *J Sports Sci* **37**, 1257-1264, doi:10.1080/02640414.2018.1554615 (2019).

65 Higgins, S., Fedewa, M. V., Hathaway, E. D., Schmidt, M. D. & Evans, E. M. Sprint interval and moderate-intensity cycling training differentially affect adiposity and aerobic capacity in overweight young-adult women. *Appl Physiol Nutr Metab* **41**, 1177-1183, doi:10.1139/apnm-2016-0240 (2016).

66 Robinson, E. *et al.* Short-term high-intensity interval and moderate-intensity continuous training reduce leukocyte TLR4 in inactive adults at elevated risk of type 2 diabetes. *J Appl Physiol (1985)* **119**, 508-516, doi:10.1152/japplphysiol.00334.2015 (2015).

67 Vella, C. A., Taylor, K. & Drummer, D. High-intensity interval and moderate-intensity continuous training elicit similar enjoyment and adherence levels in overweight and obese adults. *Eur J Sport Sci* **17**, 1203-1211, doi:10.1080/17461391.2017.1359679 (2017).

68 Blake, A., Miller, W. C. & Brown, D. A. Adiposity does not hinder the fitness response to exercise training in obese women. *J Sports Med Phys Fitness* **40**, 170-177 (2000).

69 Gondim, O. S. *et al.* Benefits of Regular Exercise on Inflammatory and Cardiovascular Risk Markers in Normal Weight, Overweight and Obese Adults. *PLoS One* **10**, e0140596, doi:10.1371/journal.pone.0140596 (2015).
70 Chen, H. T., Chung, Y. C., Chen, Y. J., Ho, S. Y. & Wu, H. J. Effects of Different Types of Exercise on Body Composition, Muscle Strength, and IGF-1 in the Elderly with Sarcopenic Obesity. *J Am Geriatr Soc* **65**, 827-832, doi:10.1111/jgs.14722 (2017).
71 Sarsan, A., Ardic, F., Ozgen, M., Topuz, O. & Sermez, Y. The effects of aerobic and resistance exercises in obese women. *Clin Rehabil* **20**, 773-782, doi:10.1177/0269215506070795 (2006).
72 Fritz, N. B. *et al.* Positive Effects of a Short-Term Intense Elastic Resistance Training Program on Body Composition and Physical Functioning in Overweight Older Women. *Biol Res Nurs* **20**, 321-334, doi:10.1177/1099800418757676 (2018).
73 Liao, C. D. *et al.* Effects of elastic band exercise on lean mass and physical capacity in older women with sarcopenic obesity: A randomized controlled trial. *Sci Rep* **8**, 2317, doi:10.1038/s41598-018-20677-7 (2018).
74 Plotnikoff, R. C. *et al.* Multicomponent, home-based resistance training for obese adults with type 2 diabetes: a randomized controlled trial. *Int J Obes (Lond)* **34**, 1733-1741, doi:10.1038/ijo.2010.109 (2010).
75 Rustaden, A. M., Haakstad, L. A. H., Paulsen, G. & Bo, K. Effects of BodyPump and resistance training with and without a personal trainer on muscle strength and body composition in overweight and obese women-A randomised controlled trial. *Obes Res Clin Pract* **11**, 728-739, doi:10.1016/j.orcp.2017.03.003 (2017).
76 Vasconcelos, K. S. *et al.* Effects of a progressive resistance exercise program with high-speed component on the physical function of older women with sarcopenic obesity: a randomized controlled trial. *Braz J Phys Ther* **20**, 432-440, doi:10.1590/bjpt-rbf.2014.0174 (2016).
77 Vincent, K. R., Braith, R. W. & Vincent, H. K. Influence of resistance exercise on lumbar strength in older, overweight adults. *Arch Phys Med Rehabil* **87**, 383-389, doi:10.1016/j.apmr.2005.11.030 (2006).
78 Wong, A. & Figueroa, A. The Effects of Low Intensity Resistance Exercise on Cardiac Autonomic Function and Muscle Strength in Obese Postmenopausal Women. *J Aging Phys Act* **27**, 855-860, doi:10.1123/japa.2018-0418 (2019).
79 Kang, H., Lee, Y., Park, D. & Kang, D. Effects of 12-week circuit weight training and aerobic exercise on body composition, physical fitness, and pulse wave velocity in obese collegiate women. *Soft Computing* **16**, 403-410 (2012).
80 Park, J., Kwon, Y. & Park, H. Effects of 24-Week Aerobic and Resistance Training on Carotid Artery Intima-Media Thickness and Flow Velocity in Elderly Women with Sarcopenic Obesity. *J Atheroscler Thromb* **24**, 1117-1124, doi:10.5551/jat.39065 (2017).
81 Zemkova, E. *et al.* Upper and Lower Body Muscle Power Increases After 3-Month Resistance Training in Overweight and Obese Men. *Am J Mens Health* **11**, 1728-1738, doi:10.1177/1557988316662878 (2017).
82 Pescatello, L. S. *et al.* The muscle strength and size response to upper arm, unilateral resistance training among adults who are overweight and obese. *J Strength Cond Res* **21**, 307-313, doi:10.1519/R-22236.1 (2007).
83 Manini, T. M. *et al.* Effects of exercise on mobility in obese and nonobese older adults. *Obesity (Silver Spring)* **18**, 1168-1175, doi:10.1038/oby.2009.317 (2010).

84 Ozaki, H., Loenneke, J. P., Thiebaud, R. S. & Abe, T. Resistance training induced increase in VO2max in young and older subjects. *Eur Rev Aging Phys Act* **10**, 107-116 (2013).

85 Zouhal, H. *et al.* Effects of physical training on anthropometrics, physical and physiological capacities in individuals with obesity: A systematic review. *Obes Rev* **21**, e13039, doi:10.1111/obr.13039 (2020).

86 Garber, C. E. *et al.* American College of Sports Medicine position stand. Quantity and quality of exercise for developing and maintaining cardiorespiratory, musculoskeletal, and neuromotor fitness in apparently healthy adults: guidance for prescribing exercise. *Med Sci Sports Exerc* **43**, 1334-1359, doi:10.1249/MSS.0b013e318213fefb (2011).

87 Sawyer, B. J. *et al.* Effects of high-intensity interval training and moderate-intensity continuous training on endothelial function and cardiometabolic risk markers in obese adults. *J Appl Physiol (1985)* **121**, 279-288, doi:10.1152/japplphysiol.00024.2016 (2016).

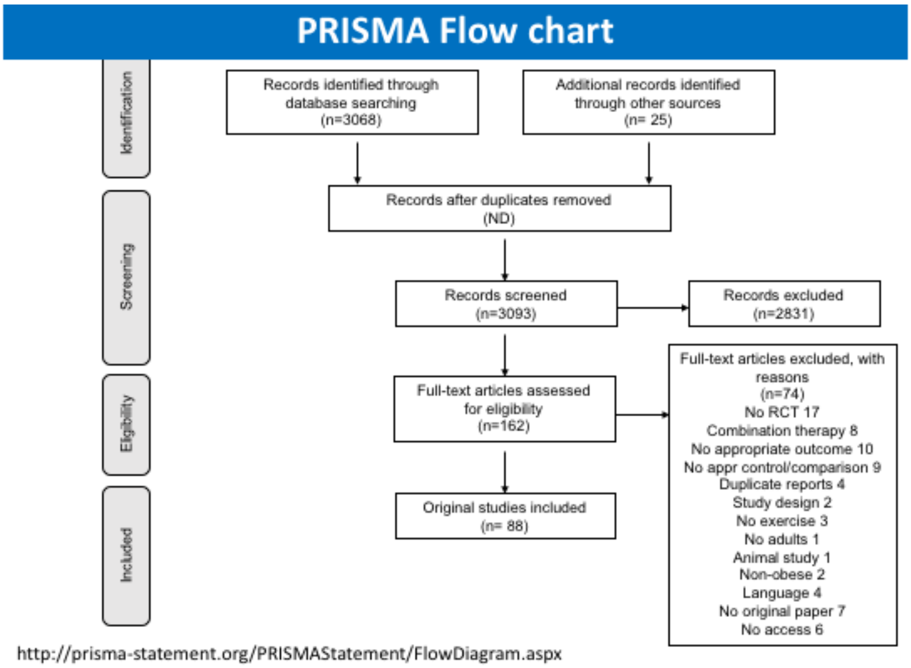

Figure S1. Flow chart of literature search.

ND = not done

Study or Subgroup	aerobic training Mean	SD	Total	no exercise Mean	SD	Total	Weight	Mean Difference IV, Random, 95% CI
Ahmadizad 2007	7	3.5	8	-2.5	6.8	8	1.8%	9.50 [4.20, 14.80]
Blond 2019	4.2	5.2	34	-0.72	4.2	6	2.7%	4.92 [1.13, 8.71]
Blond2 2019	2.68	4.4	39	-0.72	4.2	6	2.9%	3.40 [-0.23, 7.03]
Blond3 2019	5.1	5	38	-0.72	4.2	6	2.8%	5.82 [2.10, 9.54]
Brooker 2019	5.2	7.3	8	-0.7	4.4	2	1.0%	5.90 [-2.02, 13.82]
Brooker2 2009	4.6	8	7	-0.7	4.4	1	0.6%	5.30 [-5.16, 15.76]
Cao 2019	4.1	5.8	13	-1.6	6.5	15	2.2%	5.70 [1.14, 10.26]
Chin 2020	4.3	7.3	9	-0.2	5.5	14	1.7%	4.50 [-1.07, 10.07]
Church 2007	0.7	2.51	155	-0.1	2.52	34	5.4%	0.80 [-0.13, 1.73]
Church2 2007	1.5	2.09	104	-0.1	2.52	34	5.4%	1.60 [0.66, 2.54]
Church3 2007	0.8	2.51	103	-0.1	2.52	34	5.4%	0.90 [-0.08, 1.88]
Donnelly 2013	5.8	6.23	18	-1.3	4.81	5	1.9%	7.10 [2.00, 12.20]
Donnelly2 2013	6.1	9.34	19	-0.4	4.81	4	1.4%	6.50 [0.19, 12.81]
Donnelly3 2013	7.8	9.96	18	-1.3	4.81	5	1.4%	9.10 [2.86, 15.34]
Donnelly4 2013	5.6	5.83	18	-0.4	4.81	4	1.8%	6.00 [0.57, 11.43]
Duscha 2005	1.8	2.4	25	0.1	5.8	13	3.1%	1.70 [-1.59, 4.99]
Duscha2 2005	3.2	5.13	36	0.1	5.8	12	2.8%	3.10 [-0.58, 6.78]
Duscha3 2005	5.4	8.53	35	0.1	5.8	12	2.4%	5.30 [0.97, 9.63]
Emerenziani 2014	2.6	4.7	15	-0.7	5.12	15	3.0%	3.30 [-0.22, 6.82]
Gerosa-Neto 2019	1.95	4.37	13	-0.14	3.86	6	2.7%	2.09 [-1.81, 5.99]
Ho 2012	1.4	3.93	15	-2.3	4.87	16	3.3%	3.70 [0.59, 6.81]
Irving 2008	1.8	3.15	11	-0.7	3.63	4	2.6%	2.50 [-1.51, 6.51]
Irving2 2008	3	4.37	9	-0.7	3.63	3	2.0%	3.70 [-1.30, 8.70]
Jurio-Iriarte 2018	1.3	7.2	6	1.3	11.2	6	0.6%	0.00 [-10.65, 10.65]
Jurio-Iriarte2 2018	4.6	5.95	10	-2	7	8	1.5%	6.60 [0.51, 12.69]
Jurio-Iriarte3 2018	5.2	6.06	5	0.9	6.4	9	1.3%	4.30 [-2.46, 11.06]
Keating 2015	3	5.23	12	-0.4	6.07	4	1.3%	3.40 [-3.24, 10.04]
Keating2 2015	2.3	3.17	12	-0.4	6.07	4	1.5%	2.70 [-3.51, 8.91]
Keating3 2015	2.2	4.2	12	-0.4	6.07	4	1.4%	2.60 [-3.81, 9.01]
Kim 2016	3	2.92	10	-2.2	6.8	8	1.9%	5.20 [0.15, 10.25]
Kirk 2003	9.3	4.75	16	3.6	5.8	15	2.8%	5.70 [1.95, 9.45]
Kirk2 2003	6.1	3.8	25	1.3	3.5	18	4.2%	4.80 [2.60, 7.00]
Moghadasi 2011	4	3.55	8	0.11	5.7	8	2.2%	3.89 [-0.76, 8.54]
Nader 2016	6.08	2.98	10	-0.1	2.5	10	4.0%	6.18 [3.77, 8.59]
Ramos 2019	8.8	14.1	10	2.2	3.8	8	0.8%	6.60 [-2.53, 15.73]
Rayes 2019	0.2	4.35	21	-0.7	5.09	17	3.4%	0.90 [-2.15, 3.95]
Reichkendler 2012	7.7	9.17	18	1.2	4.46	9	1.9%	6.50 [1.36, 11.64]
Reichkendler2 2012	7.1	8.72	18	1.2	4.46	8	1.9%	5.90 [0.82, 10.98]
Schroeder 2019	7.7	10.3	16	1.9	7.7	17	1.4%	5.80 [-0.43, 12.03]
Utter 1998	3.5	3.75	21	1	4.22	22	4.0%	2.50 [0.12, 4.88]
Zhang 2015	7.7	4.03	15	-1.3	3.36	13	3.7%	9.00 [6.26, 11.74]
**Total (95% CI)**			**995**			**447**	**100.0%**	**4.08 [3.22, 4.95]**

Heterogeneity: $Tau^2 = 3.38$; $Chi^2 = 103.35$, df = 40 ($P < 0.00001$); $I^2 = 61\%$
Test for overall effect: $Z = 9.25$ ($P < 0.00001$)

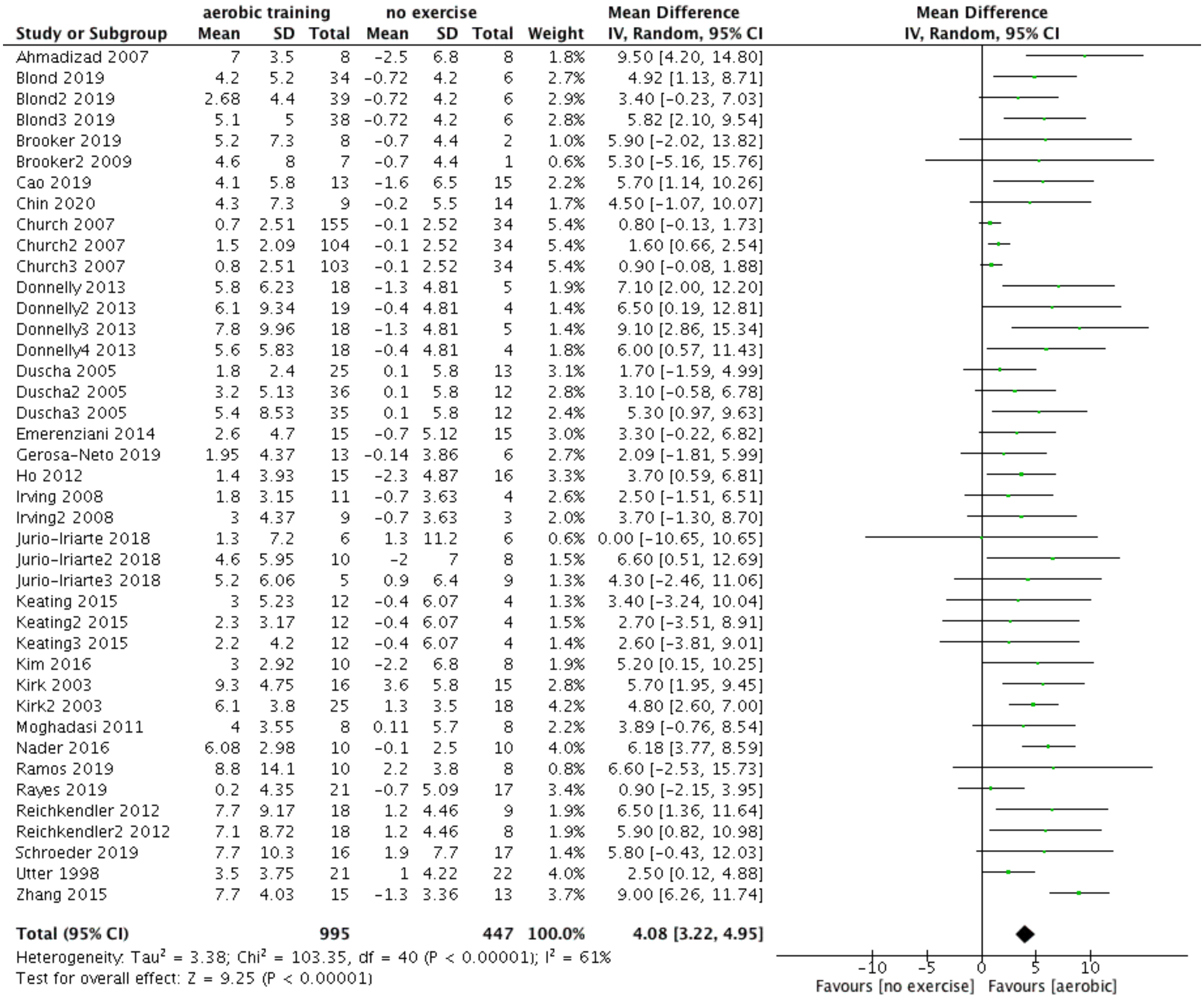

Figure S2. Forest plot of the effect of aerobic training *vs* no training on VO_{2max} in adults with overweight or obesity.

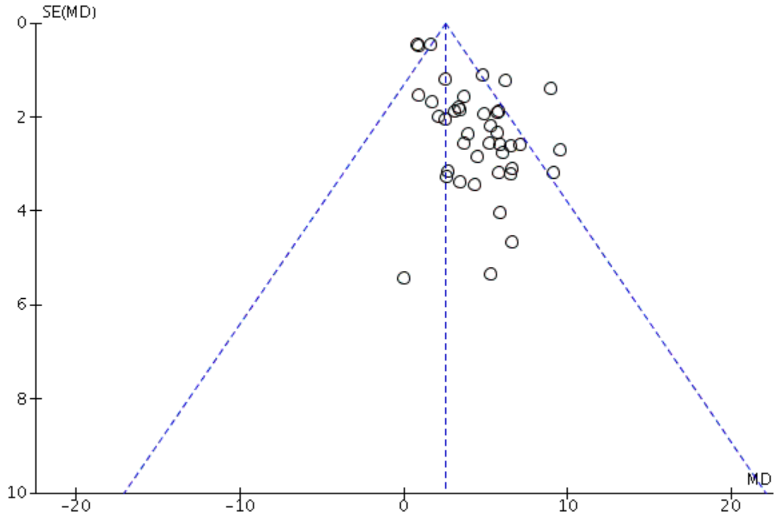

Figure S3. Funnel plot of the effect of aerobic endurance training on VO_{2max} in adults with overweight or obesity.

Study or Subgroup	resistance training Mean	SD	Total	no exercise Mean	SD	Total	Weight	Mean Difference IV, Random, 95% CI
Ahmadizad 2007	5	4.1	8	−2.5	6.8	8	13.5%	7.50 [2.00, 13.00]
Batrakoulis 2018	7.7	5.05	14	−0.3	3.2	21	21.9%	8.00 [5.02, 10.98]
Ho 2012	2	6.25	16	−2.3	4.87	16	18.6%	4.30 [0.42, 8.18]
Keating 2017	1.45	3.5	12	0.11	5.6	14	19.8%	1.34 [−2.20, 4.88]
Kim 2016	3.3	4.61	10	−2.2	6.8	8	13.5%	5.50 [−0.01, 11.01]
Schroeder 2019	1.5	8.9	15	1.9	7.7	17	12.8%	−0.40 [−6.20, 5.40]
**Total (95% CI)**			**75**			**84**	**100.0%**	**4.52 [1.76, 7.28]**

Heterogeneity: $Tau^2 = 6.74$; $Chi^2 = 12.24$, df = 5 (P = 0.03); $I^2 = 59\%$

Test for overall effect: Z = 3.21 (P = 0.001)

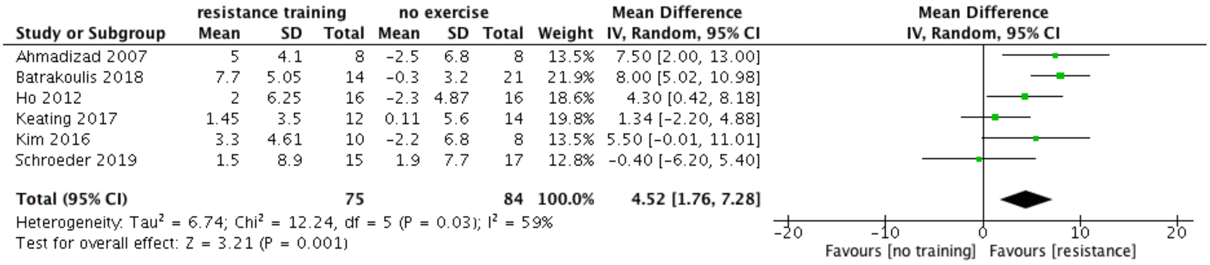

Figure S4. Forest plot of the effect of resistance training *vs* no training on VO_{2max} in adults with overweight or obesity.

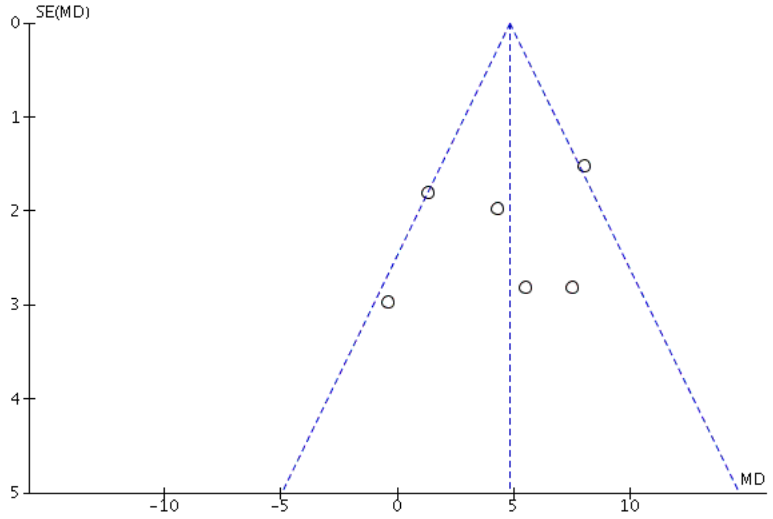

Figure S5. Funnel plot of the effect of resistance training *vs* no training on VO_{2max} in adults with overweight or obesity.

Study or Subgroup	aerobic plus resistance Mean	SD	Total	no exercise Mean	SD	Total	Weight	Mean Difference IV, Random, 95% CI
Banitalebi 2019	3.72	5.73	14	2.07	6.21	7	10.4%	1.65 [−3.84, 7.14]
Bonfante 2017	3.06	3.77	12	0.44	4.31	10	15.5%	2.62 [−0.80, 6.04]
Hara 2005	10.5	4.66	7	−3.5	4.4	7	12.0%	14.00 [9.25, 18.75]
Ho 2012	1.7	3.31	17	−2.3	4.87	16	17.0%	4.00 [1.14, 6.86]
Irwin 2003	2.72	4	87	0.05	3.1	86	21.4%	2.67 [1.60, 3.74]
Park 2015	4.59	4.47	10	−0.69	3.54	10	15.1%	5.28 [1.75, 8.81]
Schroeder 2019	4.9	11.6	18	1.9	7.7	17	8.6%	3.00 [−3.49, 9.49]
**Total (95% CI)**			**165**			**153**	**100.0%**	**4.57 [2.14, 7.00]**

Heterogeneity: $Tau^2 = 6.90$; $Chi^2 = 22.79$, df = 6 (P = 0.0009); $I^2 = 74\%$
Test for overall effect: Z = 3.69 (P = 0.0002)

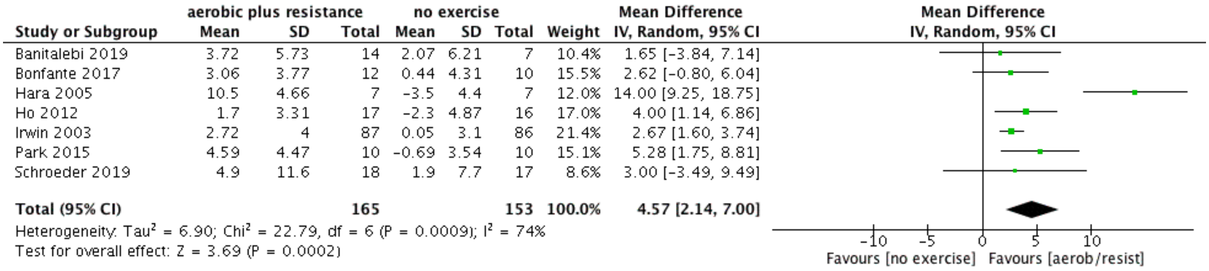

Figure S6. Forest plot of the effect of aerobic plus resistance training *vs* no training on VO_{2max} in adults with overweight or obesity.

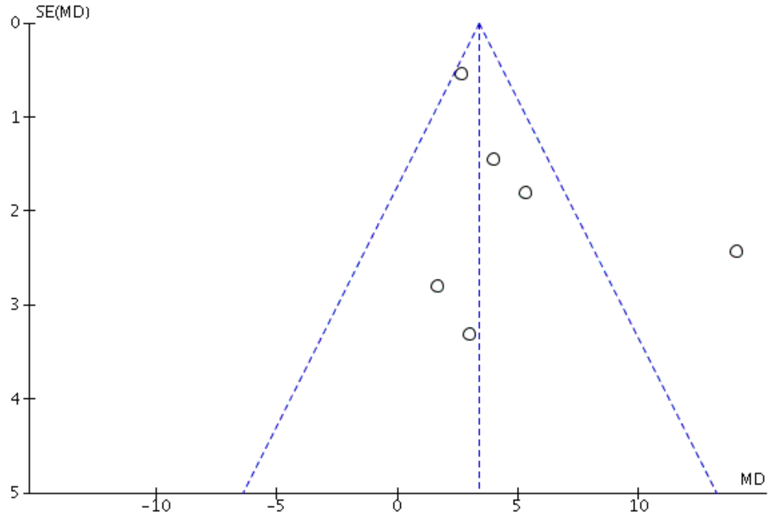

Figure S7. Funnel plot of the effect of resistance training *vs* no training on VO_{2max} in adults with overweight or obesity.

Study or Subgroup	HITT Mean	HITT SD	HITT Total	no exercise Mean	no exercise SD	no exercise Total	Weight	Mean Difference IV, Random, 95% CI
Arad 2015	1.8	5.2	9	1.2	5.6	11	5.8%	0.60 [−4.14, 5.34]
Banitalebi 2019	7.44	5.92	14	2.07	6.21	7	4.8%	5.37 [−0.18, 10.92]
Chin 2020	6.9	4.6	14	−0.2	5.5	5	4.9%	7.10 [1.71, 12.49]
Chin2 2020	6.5	6.1	10	−0.2	5.5	5	4.2%	6.70 [0.57, 12.83]
Chin3 2020	6	4.8	9	−0.2	5.5	4	4.1%	6.20 [−0.04, 12.44]
Gerosa-Neto 2019	2.86	4.52	13	−0.14	3.86	6	7.0%	3.00 [−0.95, 6.95]
Jabbour 2015	0.5	2.48	12	−1.2	7.6	12	6.1%	1.70 [−2.82, 6.22]
Jurio-Iriarte 2018	1.8	9.56	6	1.3	11.2	6	1.4%	0.50 [−11.28, 12.28]
Jurio-Iriarte2 2018	4.2	5.83	8	−2	7	8	4.0%	6.20 [−0.11, 12.51]
Jurio-Iriarte3 2018	6.4	3.04	6	0.9	6.4	9	5.6%	5.50 [0.66, 10.34]
Smith-Ryan 2016	1.8	1.86	11	0.61	2.18	5	10.6%	1.19 [−1.01, 3.39]
Smith-Ryan2 2016	2.97	1.95	10	0.61	2.18	4	10.1%	2.36 [−0.09, 4.81]
Tong 2018	7.8	10.6	16	−0.6	3.05	7	4.6%	8.40 [2.74, 14.06]
Tong2 2018	4.1	5.57	16	−0.6	3.05	7	7.8%	4.70 [1.16, 8.24]
Trilk 2010	2.9	4.12	14	−0.1	3.2	14	9.5%	3.00 [0.27, 5.73]
Zhang 2015	7.7	4.03	15	−1.3	3.36	13	9.5%	9.00 [6.26, 11.74]
**Total (95% CI)**			**183**			**123**	**100.0%**	**4.31 [2.81, 5.80]**

Heterogeneity: $Tau^2 = 4.18$; $Chi^2 = 30.85$, df = 15 (P = 0.009); $I^2 = 51\%$
Test for overall effect: Z = 5.65 (P < 0.00001)

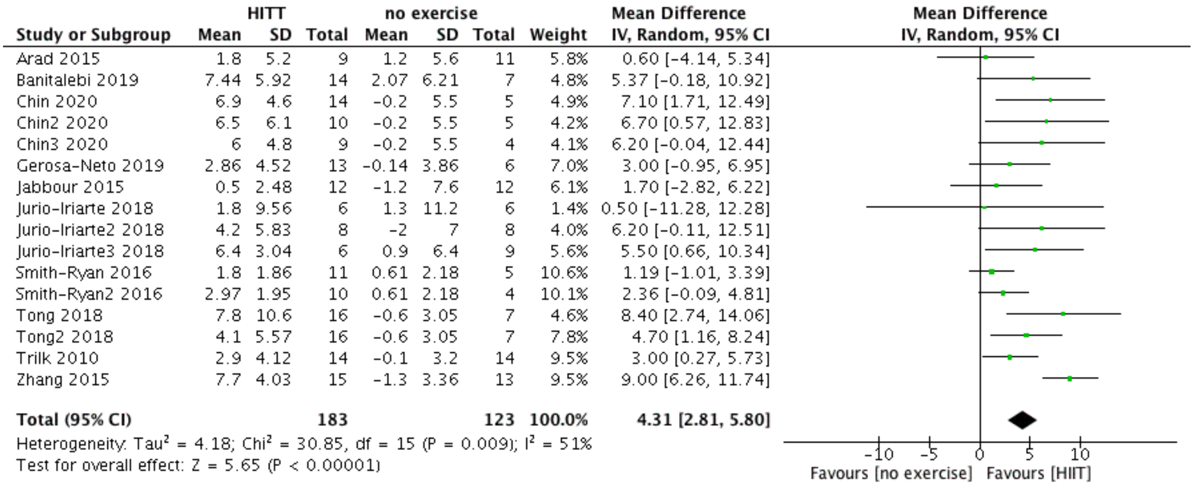

Figure S8. Forest plot of the effect of high-intensity interval training (HIIT) *vs* no training on VO_{2max} in adults with overweight or obesity.

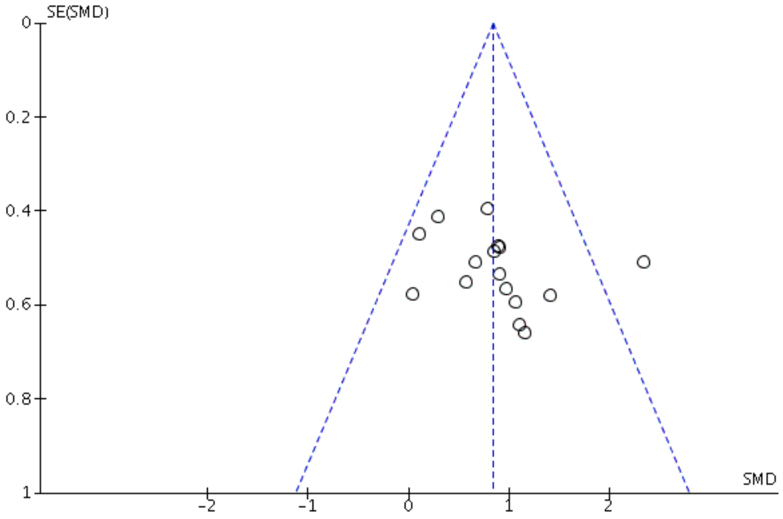

Figure S9. Funnel plot of the effect of high-intensity interval training (HIIT) on VO_{2max} in adults with overweight or obesity.

Study or Subgroup	resistance exercise Mean	SD	Total	aerobic exercise Mean	SD	Total	Weight	Mean Difference IV, Random, 95% CI
Ahmadizad 2007	5	4.1	8	7	3.5	8	6.9%	−2.00 [−5.74, 1.74]
Ho 2012	2	6.25	16	1.4	3.93	15	7.2%	0.60 [−3.05, 4.25]
Kim 2016	3.3	4.61	10	3.1	2.92	10	8.3%	0.20 [−3.18, 3.58]
Kim 2020	3.34	4.32	14	5.28	4.13	13	9.2%	−1.94 [−5.13, 1.25]
Sawczyn 2015	2.54	3.02	15	2.31	3.14	14	17.1%	0.23 [−2.02, 2.48]
Schjerve 2008	2.7	5.8	13	4.2	5	13	5.6%	−1.50 [−5.66, 2.66]
Schroeder 2019	1.5	8.9	15	7.7	10.3	16	2.2%	−6.20 [−12.96, 0.56]
Slentz 2011	1.4	3	52	3.6	3	48	43.5%	−2.20 [−3.38, −1.02]
**Total (95% CI)**			**143**			**137**	**100.0%**	**−1.40 [−2.41, −0.38]**

Heterogeneity: $Tau^2 = 0.26$; $Chi^2 = 7.87$, df = 7 (P = 0.34); $I^2 = 11\%$
Test for overall effect: Z = 2.70 (P = 0.007)

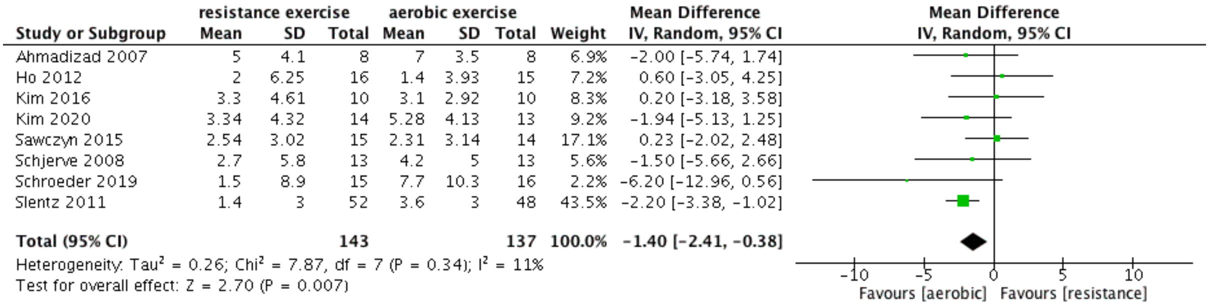

Figure S10. Forest plot comparing the effects of resistance training with aerobic training on VO_{2max} in adults with overweight or obesity.

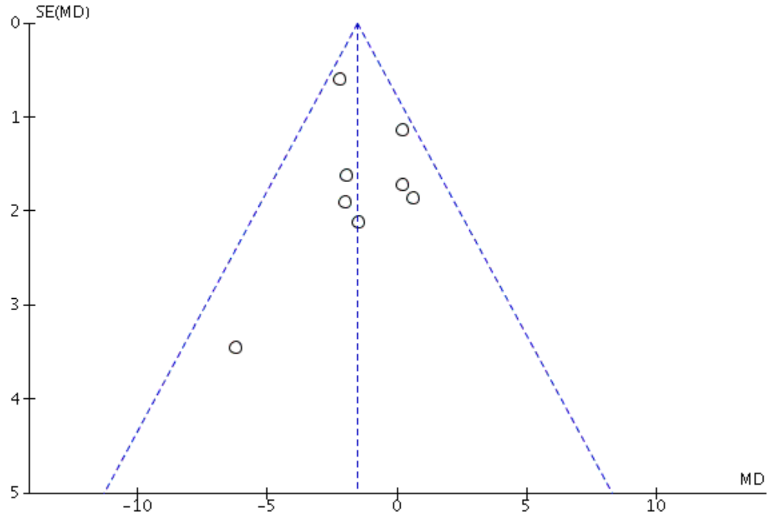

Figure S11. Funnel plot of studies comparing the effects of aerobic and resistance training on VO_{2max} in adults with overweight or obesity.

	Aerobic plus resistance			Aerobic exercise				Mean Difference
Study or Subgroup	Mean	SD	Total	Mean	SD	Total	Weight	IV, Random, 95% CI
Ho 2012	1.7	3.31	17	1.4	3.93	15	15.8%	0.30 [−2.24, 2.84]
Schroeder 2019	4.9	11.6	18	7.7	10.3	16	1.9%	−2.80 [−10.16, 4.56]
Skrypnik 2015	3.8	3.9	17	3.1	3.69	21	17.1%	0.70 [−1.73, 3.13]
Slentz 2011	4	3	44	3.6	3	45	65.3%	0.40 [−0.85, 1.65]
**Total (95% CI)**			**96**			**97**	**100.0%**	**0.38 [−0.63, 1.38]**

Heterogeneity: Tau² = 0.00; Chi² = 0.79, df = 3 (P = 0.85); I² = 0%
Test for overall effect: Z = 0.73 (P = 0.46)

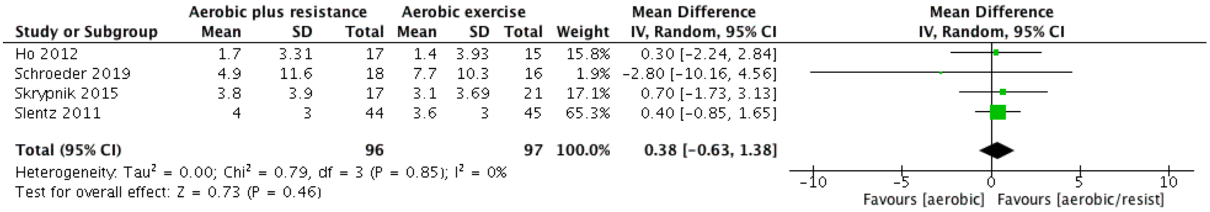

Figure S12. Forest plot comparing the effects of combined aerobic plus resistance training with aerobic training on VO_{2max} in adults with overweight or obesity.

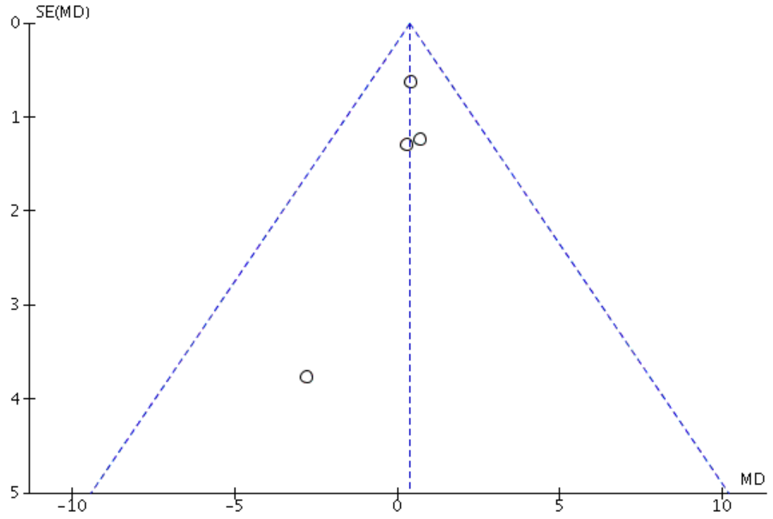

Figure S13. Funnel plot of studies comparing the effects of combined aerobic plus resistance training and aerobic training on VO_{2max} in adults with overweight or obesity.

Study or Subgroup	HIIT Mean	HIIT SD	HIIT Total	aerobic exercise Mean	aerobic exercise SD	aerobic exercise Total	Weight	Mean Difference IV, Random, 95% CI
Baekkerud 2016	2.8	7.95	8	0.4	6.13	5	0.9%	2.40 [-5.30, 10.10]
Baekkerud2 2016	0.9	7.35	9	0.4	6.13	4	0.9%	0.50 [-7.19, 8.19]
Cheema 2015	4.6	3.1	6	-0.2	1.66	6	6.8%	4.80 [1.99, 7.61]
Chin 2020	6.9	4.6	14	4.3	7.3	3	0.7%	2.60 [-6.00, 11.20]
Chin2 2020	6.5	6.1	10	4.3	7.3	3	0.7%	2.20 [-6.88, 11.28]
Chin3 2020	6	4.8	9	4.3	7.3	3	0.7%	1.70 [-7.14, 10.54]
Cocks 2016	2.4	4.08	8	4.7	6.63	8	1.9%	-2.30 [-7.69, 3.09]
De Strijcker 2018	2	7.35	8	0.2	9.61	8	0.8%	1.80 [-6.58, 10.18]
Fisher 2015	0.84	3.5	13	3.4	3.36	10	6.8%	-2.56 [-5.38, 0.26]
Gerosa-Neto 2019	2.86	4.52	13	1.95	4.37	13	4.6%	0.91 [-2.51, 4.33]
Higgins 2016	4.1	6.01	29	1.9	2.4	23	9.4%	2.20 [-0.20, 4.60]
Jurio-Iriarte 2018	1.8	9.56	6	1.3	7.2	6	0.6%	0.50 [-9.08, 10.08]
Jurio-Iriarte2 2018	4.2	5.83	8	4.6	5.95	10	1.8%	-0.40 [-5.87, 5.07]
Jurio-Iriarte3 2018	6.4	3.04	6	5.2	6.06	5	1.6%	1.20 [-4.64, 7.04]
Kong 2016	2.5	6.2	10	4	5.73	8	1.8%	-1.50 [-7.03, 4.03]
Lunt 2014	1.4	4.8	9	-1.3	4.69	7	2.5%	2.70 [-1.98, 7.38]
Lunt2 2014	0.2	3.14	9	-1.3	4.69	7	3.3%	1.50 [-2.53, 5.53]
Martins 2016	2.8	2.6	16	2.9	2.9	7	8.7%	-0.10 [-2.60, 2.40]
Martins2 2016	4.4	2.2	16	2.9	2.9	7	9.4%	1.50 [-0.90, 3.90]
Robinson 2015	1.5	3.74	20	1.5	4.8	18	7.1%	0.00 [-2.76, 2.76]
Sawyer 2016	4.1	3.31	8	3.1	2.77	9	6.3%	1.00 [-1.92, 3.92]
Schjerve 2008	8	5.3	14	4.2	5	13	3.6%	3.80 [-0.09, 7.69]
Sun 2019	7.8	3.1	14	7.8	3.4	7	6.0%	0.00 [-3.00, 3.00]
Sun2 2019	8.4	4.1	14	7.8	3.4	7	4.9%	0.60 [-2.71, 3.91]
Vella 2017	2.6	7.38	8	0.4	5.51	9	1.4%	2.20 [-4.05, 8.45]
Zhang 2015	8.4	3.9	15	7.7	4.03	15	6.7%	0.70 [-2.14, 3.54]
**Total (95% CI)**			**300**			**221**	**100.0%**	**0.99 [0.25, 1.73]**

Heterogeneity: $Tau^2 = 0.00$; $Chi^2 = 21.63$, df = 25 (P = 0.66); $I^2 = 0\%$

Test for overall effect: Z = 2.64 (P = 0.008)

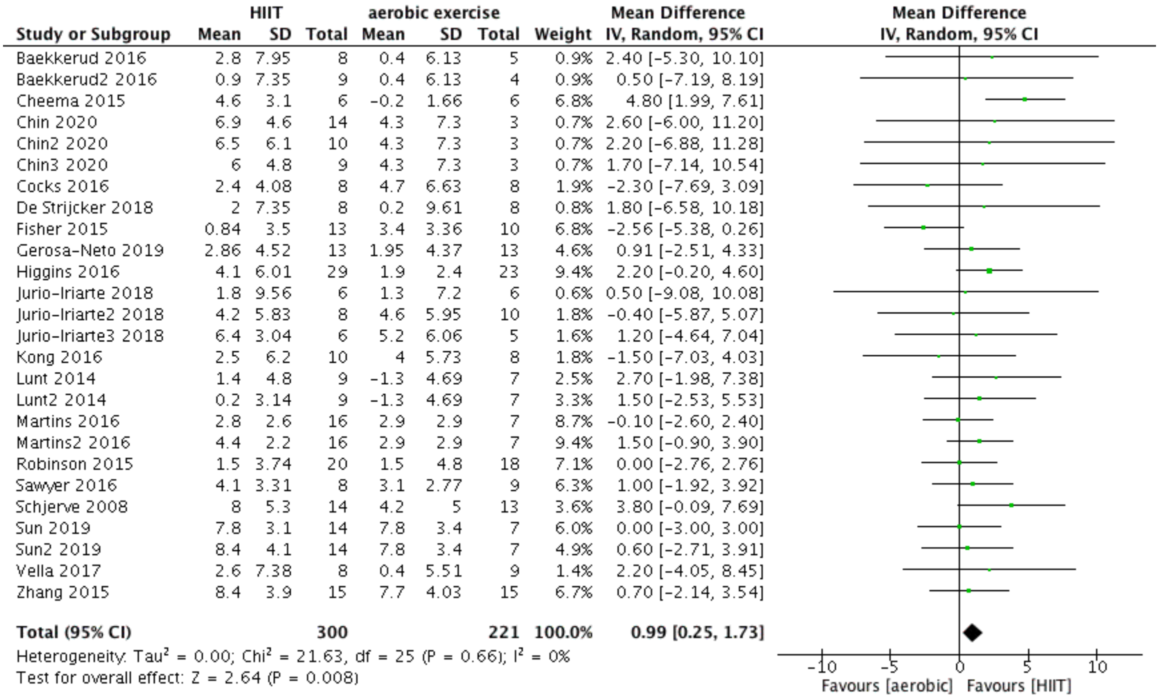

Figure S14. Forest plot comparing the effects of HIIT with aerobic training on VO_{2max} in adults with overweight or obesity.

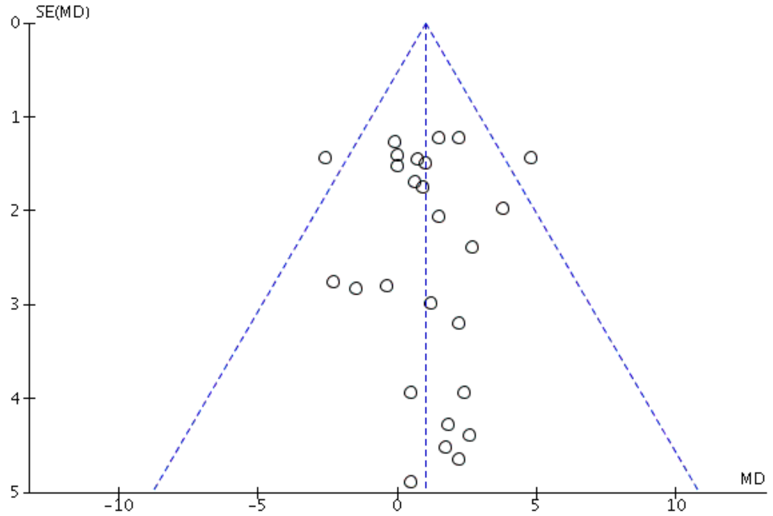

Figure S15. Funnel plot of studies comparing the effects of HIIT and aerobic training on VO_{2max} in adults with overweight or obesity.

Study or Subgroup	Aerobic exercise Mean	SD	Total	No exercise Mean	SD	Total	Weight	Std. Mean Difference IV, Random, 95% CI
Cao 2019	2.4	5.13	7	−1.1	5.03	8	9.4%	0.65 [−0.40, 1.70]
Chen 2017	−2.9	7.19	5	−2.5	9.31	5	6.8%	−0.04 [−1.28, 1.20]
Chen2 2017	7.82	22.02	5	−2.86	24.41	5	6.5%	0.41 [−0.85, 1.68]
Chen3 2017	−0.43	6.99	5	−1.47	7.74	5	6.7%	0.13 [−1.11, 1.37]
Kim 2016	−0.1	10.55	4	−0.5	8.78	3	4.6%	0.03 [−1.46, 1.53]
Kim2 2016	−3.9	45.52	3	−7.2	60.46	3	4.1%	0.05 [−1.55, 1.65]
Kim3 2016	16	24.88	3	2.3	37.17	2	3.1%	0.34 [−1.51, 2.18]
Ramos 2019	0.8	1.61	10	2.1	4.63	8	11.8%	−0.38 [−1.32, 0.56]
Sarsan 2006	9.1	10.48	10	1.1	3.62	10	11.8%	0.98 [0.04, 1.92]
Sarsan2 2006	1.6	2.5	10	0.25	2.91	10	13.1%	0.48 [−0.42, 1.37]
Schroeder 2019	−1	33.78	8	2	36.95	9	11.5%	−0.08 [−1.03, 0.87]
Schroeder2 2019	4	7.51	8	2	7.78	8	10.7%	0.25 [−0.74, 1.23]
**Total (95% CI)**			**78**			**76**	**100.0%**	**0.26 [−0.06, 0.58]**

Heterogeneity: $Tau^2 = 0.00$; $Chi^2 = 5.74$, df = 11 (P = 0.89); $I^2 = 0\%$

Test for overall effect: Z = 1.57 (P = 0.12)

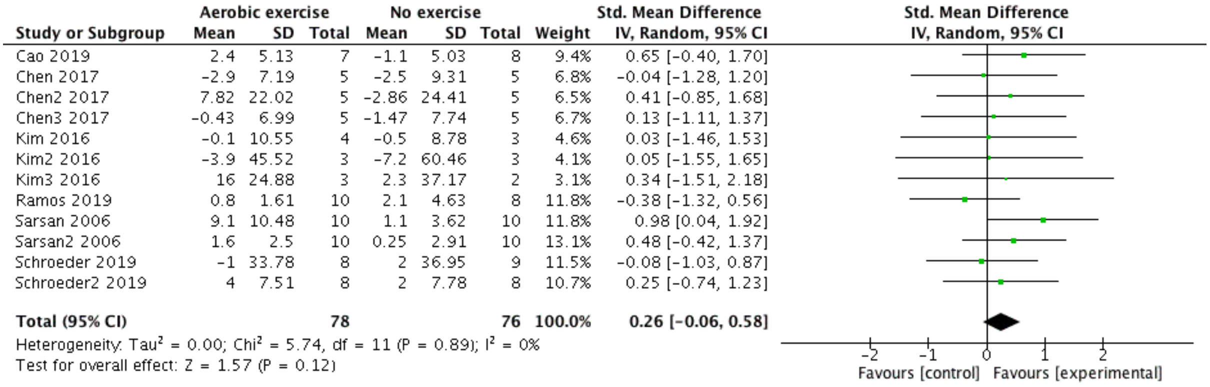

Figure S16. Forest plot comparing the effects of aerobic training vs no training on muscle strength in adults with overweight or obesity.

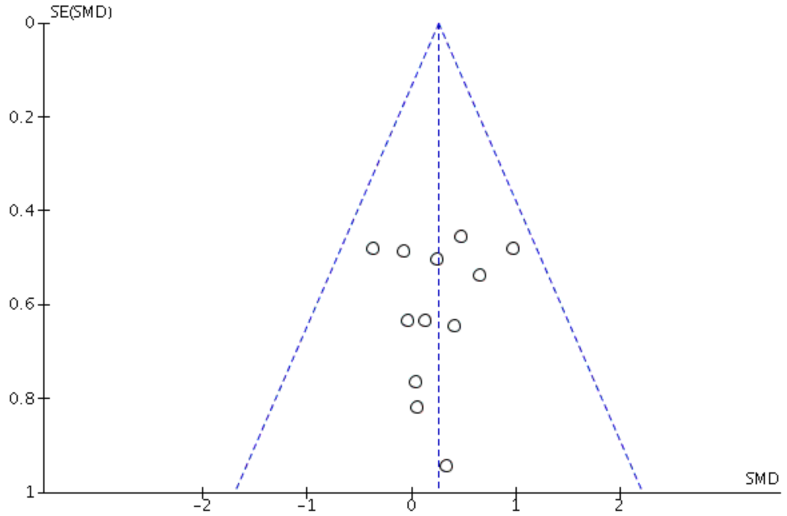

Figure S17. Funnel plot of the effect of aerobic training on muscle strength in adults with overweight or obesity.

Study or Subgroup	Resistance training Mean	SD	Total	No training Mean	SD	Total	Weight	Std. Mean Difference IV, Random, 95% CI
Batrakoulis 2018	33.9	30.1	14	1.7	28.7	21	7.0%	1.08 [0.35, 1.80]
Chen 2017	3.5	7.15	5	−2.5	9.31	5	2.2%	0.65 [−0.64, 1.95]
Chen2 2017	9.04	23.69	5	−2.86	24.41	5	2.3%	0.45 [−0.82, 1.71]
Chen3 2017	4.2	5.67	5	−1.47	7.74	5	2.2%	0.75 [−0.56, 2.07]
Fritz 2018	11.85	13.52	8	9.63	39.4	4	2.6%	0.08 [−1.12, 1.29]
Fritz2 2018	28.98	33.08	7	−1.55	9.6	3	1.8%	0.95 [−0.50, 2.40]
Fritz3 2018	8.24	9.4	7	−2.19	11.78	3	1.8%	0.94 [−0.51, 2.39]
Fritz4 2018	4.04	4.47	7	9.63	39.4	4	2.4%	−0.22 [−1.46, 1.01]
Fritz5 2018	30.18	33.36	7	−1.68	2.05	3	1.7%	1.00 [−0.47, 2.46]
Fritz6 2018	8.56	10.19	7	−0.47	1.01	3	1.8%	0.92 [−0.53, 2.37]
Kim 2016	3.5	3.41	4	−0.5	8.77	3	1.5%	0.55 [−1.01, 2.11]
Kim2 2016	23.6	23	3	−7.2	60.46	3	1.3%	0.54 [−1.14, 2.22]
Kim3 2016	12.3	24.7	3	2.3	37.2	2	1.1%	0.25 [−1.57, 2.07]
Liao 2018	2.85	1.97	17	1.68	3.71	12	6.7%	0.40 [−0.34, 1.15]
Liao2 2018	1.35	0.96	16	−0.47	1.83	11	5.1%	1.28 [0.43, 2.14]
Plotnikoff 2010	9	24.4	9	−0.1	15.85	7	3.7%	0.41 [−0.59, 1.41]
Plotnikoff2 2010	46.9	123.5	9	−5.7	62.47	6	3.4%	0.47 [−0.58, 1.53]
Plotnikoff3 2010	14	25.1	9	1.5	20.03	7	3.7%	0.51 [−0.50, 1.52]
Rustaden 2017	12.3	12	13	4	10.6	4	2.8%	0.67 [−0.48, 1.82]
Rustaden2 2017	3.8	2.6	12	1.7	4.2	4	2.8%	0.66 [−0.50, 1.82]
Rustaden3 2017	28.4	11.5	15	4	10.6	4	2.1%	2.05 [0.73, 3.38]
Rustaden4 2017	8.1	4.6	14	1.7	4.2	4	2.5%	1.35 [0.13, 2.56]
Rustaden5 2017	14.4	10.8	11	4	10.6	4	2.6%	0.91 [−0.30, 2.12]
Rustaden6 2017	7.8	3.3	10	1.7	4.2	3	1.7%	1.63 [0.14, 3.12]
Sarsan 2006	14	16.12	10	1.1	3.62	10	4.1%	1.06 [0.11, 2.01]
Sarsan2 2006	3.37	3.88	10	0.25	2.91	10	4.3%	0.87 [−0.06, 1.80]
Schroeder 2019	13	34.31	8	2	36.95	9	4.0%	0.29 [−0.67, 1.25]
Schroeder2 2019	4	7.31	7	2	7.78	8	3.6%	0.25 [−0.77, 1.27]
Vasconcelos 2016	2.36	12	14	−8.7	20	14	6.4%	0.65 [−0.11, 1.41]
Vincent 2006	143	258.2	8	−11	324.6	8	3.7%	0.50 [−0.50, 1.50]
Vincent2 2006	103	186	7	4	154	7	3.2%	0.54 [−0.53, 1.62]
Wong 2019	27.7	22.4	10	0.7	16.5	10	3.8%	1.31 [0.33, 2.30]
**Total (95% CI)**			**291**			**206**	**100.0%**	**0.74 [0.54, 0.93]**

Heterogeneity: $Tau^2 = 0.00$; $Chi^2 = 18.60$, df = 31 ($P = 0.96$); $I^2 = 0\%$
Test for overall effect: $Z = 7.48$ ($P < 0.00001$)

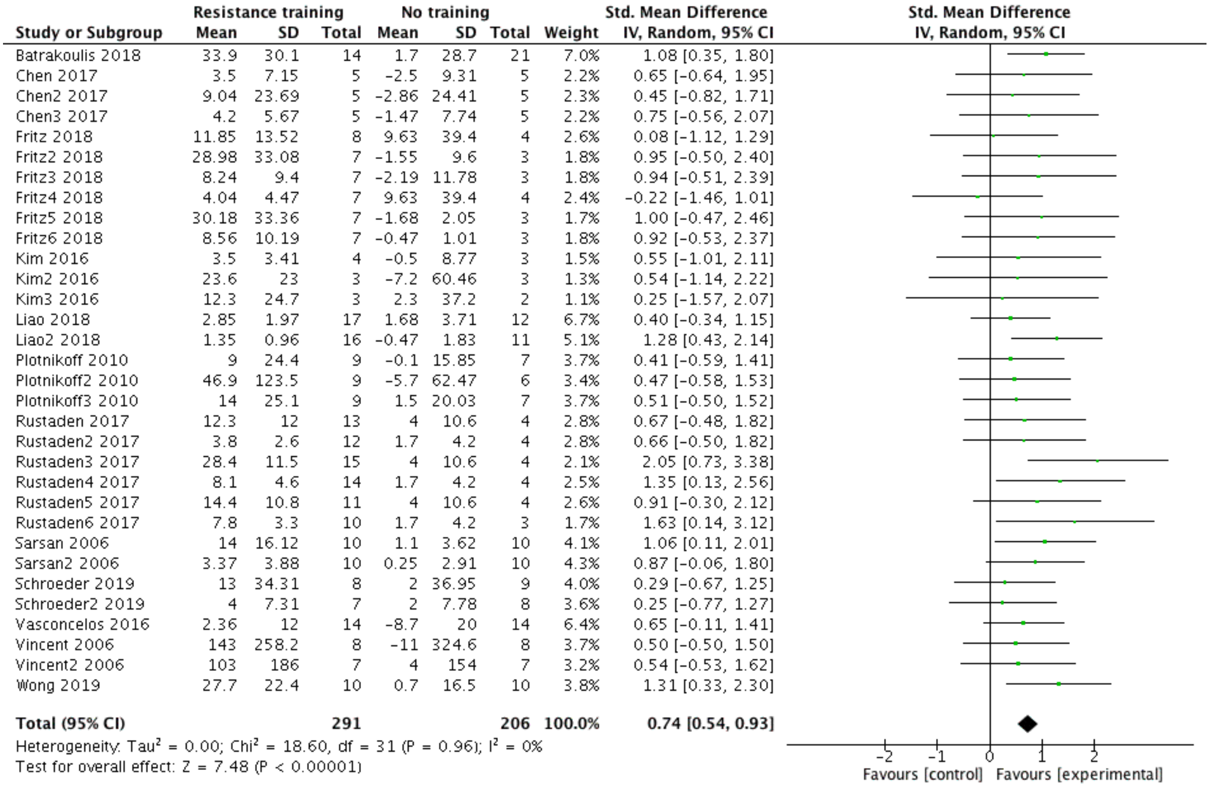

Figure S18. Forest plot comparing the effects of resistance training vs no training on muscle strength in adults with overweight or obesity.

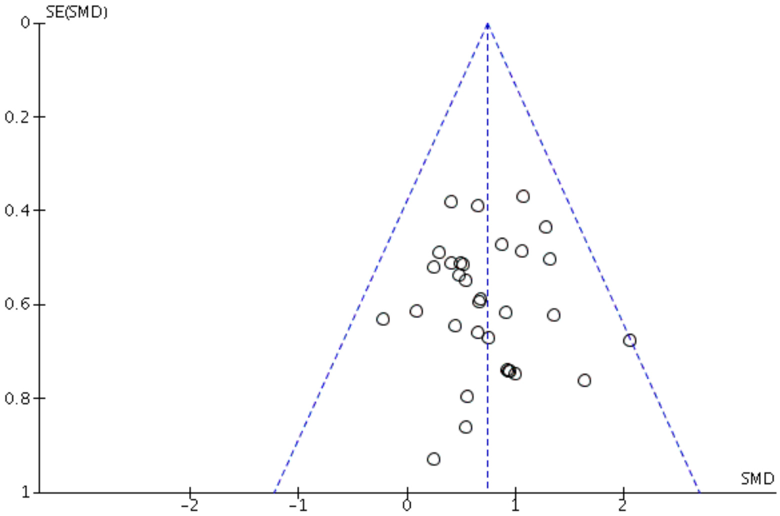

Figure S19. Funnel plot of the effect of resistance training on muscle strength in adults with overweight or obesity.

Study or Subgroup	Aerobic plus resistance Mean	SD	Total	No exercise Mean	SD	Total	Weight	Std. Mean Difference IV, Random, 95% CI
Bonfante 2017	14.36	18.57	4	−10.89	14.42	3	3.7%	1.25 [−0.55, 3.04]
Bonfante2 2017	63	69.85	4	8.89	75.5	4	5.6%	0.65 [−0.81, 2.10]
Bonfante3 2017	5.82	4.67	4	0.89	5.75	3	4.4%	0.81 [−0.82, 2.44]
Chen 2017	−1.9	7.26	5	−2.5	9.31	5	7.7%	0.06 [−1.18, 1.31]
Chen2 2017	10.02	22.31	5	−2.86	24.41	5	7.3%	0.50 [−0.77, 1.77]
Chen3 2017	1.69	7	5	−1.47	7.74	5	7.4%	0.39 [−0.87, 1.65]
Kang 2012	12.34	13.58	3	0	12.88	3	3.8%	0.75 [−1.01, 2.50]
Kang2 2012	1.77	3.7	3	−2.07	7.71	3	4.2%	0.51 [−1.17, 2.18]
Park 2015	3.52	2.88	5	−0.46	3.01	5	5.9%	1.22 [−0.20, 2.64]
Park 2017	3.2	4.27	13	−0.5	0.24	13	16.5%	1.18 [0.34, 2.03]
Park2 2015	6.26	9.7	5	−1.31	11.39	5	7.1%	0.65 [−0.65, 1.94]
Schroeder 2019	11	36.2	9	2	36.95	9	13.7%	0.23 [−0.69, 1.16]
Schroeder2 2019	4	6.03	9	2	7.78	8	12.8%	0.28 [−0.68, 1.23]
**Total (95% CI)**			**74**			**71**	**100.0%**	**0.62 [0.27, 0.96]**

Heterogeneity: $Tau^2 = 0.00$; $Chi^2 = 5.07$, df = 12 (P = 0.96); $I^2 = 0\%$

Test for overall effect: Z = 3.53 (P = 0.0004)

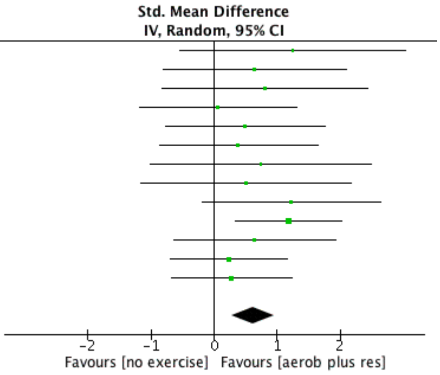

Figure S20. Forest plot comparing the effects of combined aerobic plus resistance training vs no training on muscle strength in adults with overweight or obesity.

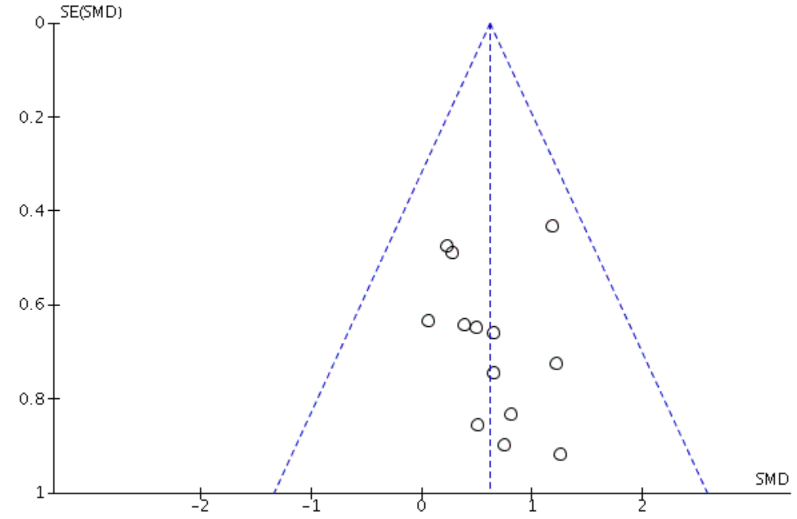

Figure S21. Funnel plot of the effect of combined aerobic plus resistance training on muscle strength in adults with overweight or obesity.

Study or Subgroup	Resistance training Mean	SD	Total	Aerobic training Mean	SD	Total	Weight	Std. Mean Difference IV, Random, 95% CI
Chen 2017	3.5	7.15	5	-2.9	7.19	5	4.9%	0.81 [-0.51, 2.13]
Chen2 2017	4.2	5.67	5	-0.43	6.99	5	5.1%	0.66 [-0.64, 1.95]
Chen3 2017	9.04	23.69	5	7.82	22.02	5	5.6%	0.05 [-1.19, 1.29]
Kim 2016	3.5	3.41	4	-0.1	10.55	4	4.3%	0.40 [-1.01, 1.81]
Kim 2020	0.18	0.22	14	-0.01	0.19	13	13.4%	0.89 [0.10, 1.69]
Kim2 2016	23.6	23	3	-3.9	45.52	3	2.9%	0.61 [-1.10, 2.32]
Kim3 2016	12.3	24.7	3	16	24.88	3	3.3%	-0.12 [-1.72, 1.49]
Sarsan 2006	14	16.12	10	5.23	9.1	10	10.4%	0.64 [-0.26, 1.55]
Sarsan2 2006	3.37	3.88	10	2.93	1.6	10	11.1%	0.14 [-0.74, 1.02]
Schjerve 2008	43.9	42.8	13	2.5	43.7	13	12.8%	0.93 [0.11, 1.74]
Schroeder 2019	13	34.3	8	-1	33.8	8	8.7%	0.39 [-0.60, 1.38]
Schroeder2 2019	4	7.3	7	4	7.5	8	8.3%	0.00 [-1.01, 1.01]
Zemkova 2017	137.7	615.1	5	-23.8	638.5	4	4.9%	0.23 [-1.09, 1.55]
Zemkova2 2017	3.9	4.63	4	2.3	4.06	4	4.3%	0.32 [-1.08, 1.72]
**Total (95% CI)**			**96**			**95**	**100.0%**	**0.49 [0.19, 0.78]**

Heterogeneity: $Tau^2 = 0.00$; $Chi^2 = 5.30$, df = 13 (P = 0.97); $I^2 = 0\%$

Test for overall effect: Z = 3.27 (P = 0.001)

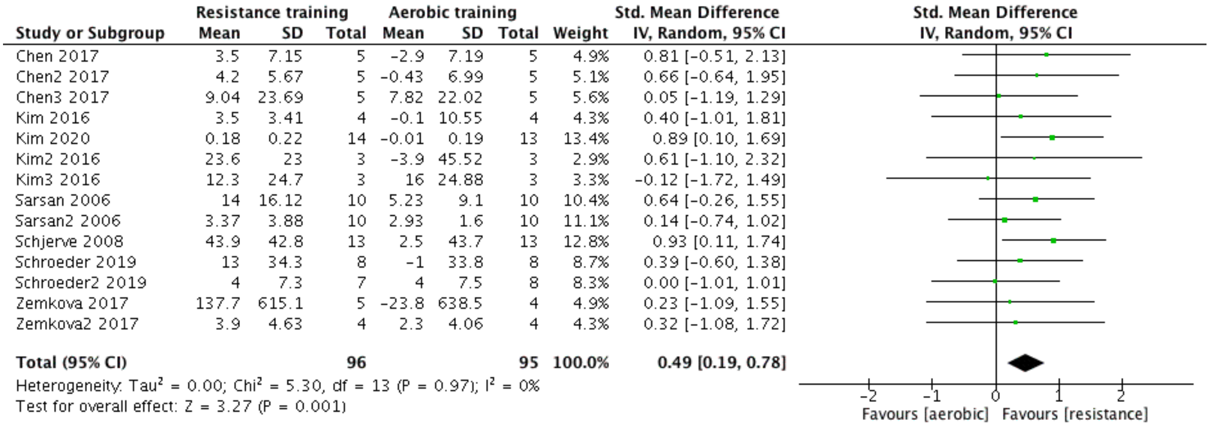

Figure S22. Forest plot comparing the effects of resistance training vs aerobic training on muscle strength in adults with overweight or obesity.

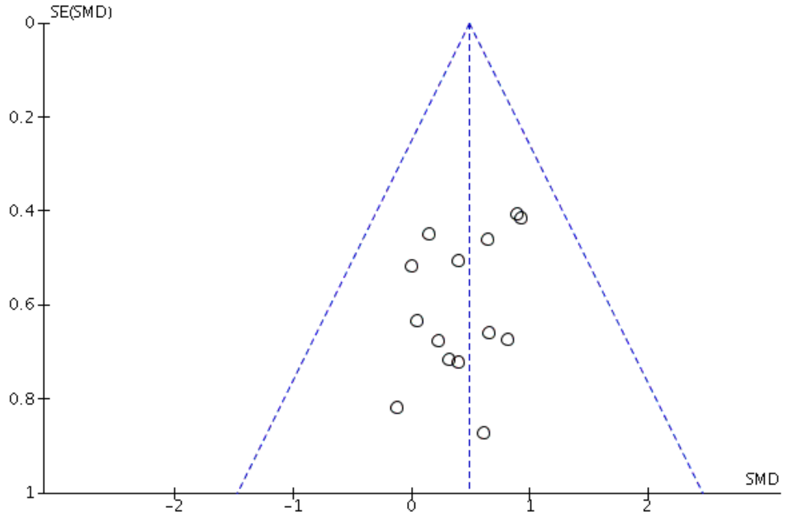

Figure S23. Funnel plot of the effect of resistance training vs aerobic training on muscle strength in adults with overweight or obesity.

Study or Subgroup	resist or aer/resist Mean	SD	Total	no exercise Mean	SD	Total	Weight	Std. Mean Difference IV, Random, 95% CI
Fritz 2018	40	81.7	4	-11.44	62.5	2	2.7%	0.53 [-1.24, 2.31]
Fritz10 2018	46	81	4	-11.44	62.5	2	2.6%	0.60 [-1.19, 2.39]
Fritz11 2018	5.95	6.79	4	-1.3	6.49	2	2.4%	0.86 [-1.03, 2.75]
Fritz12 2018	7.45	8.5	4	0.25	4.17	2	2.5%	0.75 [-1.09, 2.60]
Fritz2 2018	3.42	3.9	3	-0.11	0.56	1	0.6%	0.52 [-3.18, 4.22]
Fritz3 2018	2.7	3.08	3	-0.38	2.08	1	0.5%	0.57 [-3.38, 4.52]
Fritz4 2018	1.24	1.52	4	-0.14	1.61	2	2.5%	0.72 [-1.12, 2.55]
Fritz5 2018	6.52	7.76	4	-1.3	6.49	2	2.4%	0.84 [-1.04, 2.72]
Fritz6 2018	8.06	8.91	4	0.25	4.17	2	2.5%	0.78 [-1.08, 2.64]
Fritz7 2018	2.32	4.08	3	-0.11	0.56	2	2.3%	0.53 [-1.40, 2.45]
Fritz8 2018	2.97	4.32	3	-0.38	2.08	1	0.7%	0.44 [-2.93, 3.82]
Fritz9 2018	0.94	1.13	3	-0.14	1.61	1	0.6%	0.55 [-3.28, 4.38]
Kang 2012	4.22	4.13	3	-1.42	4.83	3	2.4%	1.00 [-0.87, 2.88]
Kang5 2012	15.5	24	3	-8.33	13.08	3	2.4%	0.99 [-0.88, 2.85]
Liao 2018	9.58	7.67	6	2.15	6.93	4	4.5%	0.91 [-0.46, 2.27]
Liao2 2018	6.69	6.68	6	-3.05	7.04	4	4.0%	1.29 [-0.17, 2.75]
Liao3 2018	0.07	0.16	6	-0.07	0.22	4	4.8%	0.68 [-0.64, 2.01]
Liao4 2018	1.67	1.18	5	0.02	1.24	4	3.7%	1.22 [-0.30, 2.73]
Liao5 2018	2.48	2.57	5	-0.51	2.69	4	4.0%	1.01 [-0.44, 2.47]
Liao6 2018	1.34	2.38	5	-2.23	2.35	3	3.0%	1.31 [-0.38, 3.00]
Park 2015	3.3	4.92	3	-0.5	4.31	3	2.9%	0.66 [-1.06, 2.38]
Park 2017	1.5	3.64	7	-1	2.7	7	7.1%	0.73 [-0.36, 1.83]
Park2 2015	4.4	3.3	3	-0.4	3.2	3	2.2%	1.18 [-0.78, 3.15]
Park2 2017	1.8	4.5	6	-0.5	4.4	6	6.3%	0.48 [-0.68, 1.63]
Park3 2015	1.06	5.3	2	-0.27	4.08	2	1.8%	0.16 [-2.00, 2.32]
Park3 2017	0.15	0.27	6	-0.04	0.07	6	5.8%	0.89 [-0.32, 2.10]
Park4 2015	0.36	5.11	2	-0.1	9.39	2	2.2%	0.03 [-1.94, 2.00]
Park4 2017	6.2	8.28	6	-1.9	4.6	6	5.4%	1.12 [-0.14, 2.37]
Vasconcelos 2016	0.4	1.3	7	0.57	1	7	7.7%	-0.14 [-1.19, 0.91]
Vasconcelos2 2016	0.02	0.01	7	0.05	0.17	7	7.6%	-0.23 [-1.29, 0.82]
**Total (95% CI)**			**131**			**98**	**100.0%**	**0.66 [0.37, 0.95]**

Heterogeneity: $Tau^2 = 0.00$; $Chi^2 = 9.19$, df = 29 ($P = 1.00$); $I^2 = 0\%$
Test for overall effect: $Z = 4.46$ ($P < 0.00001$)

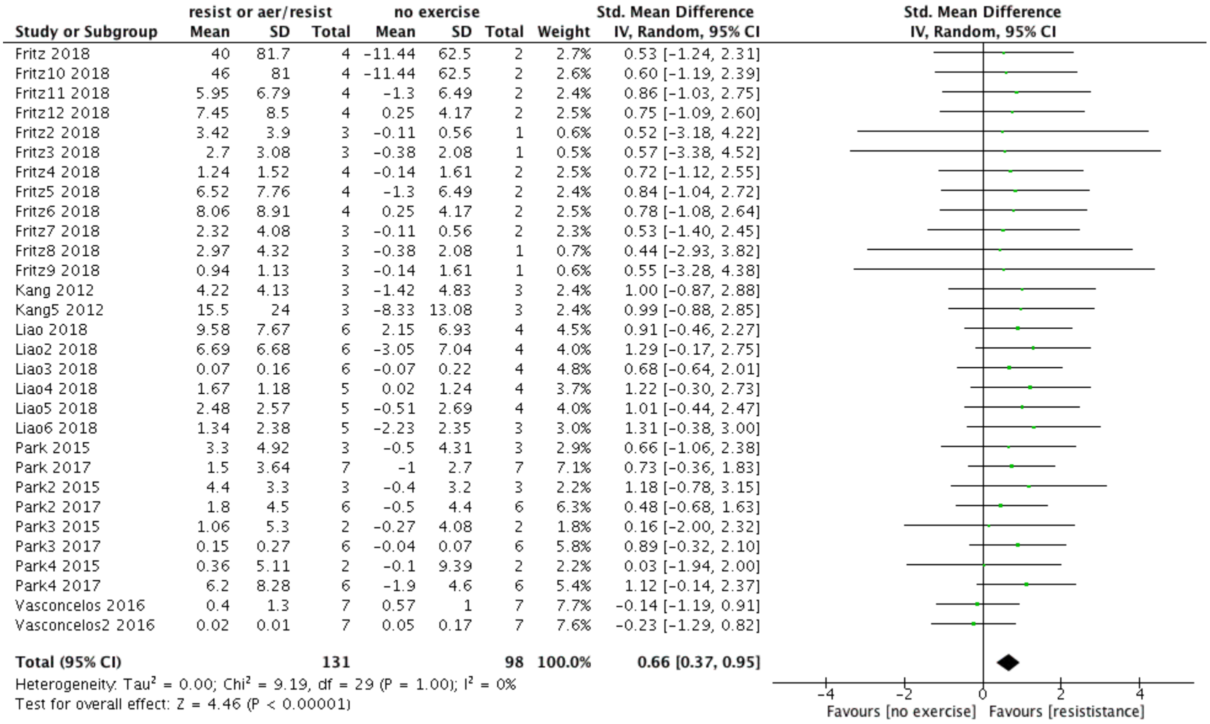

Figure S24. Forest plot of the effects of resistance training alone or in combination with aerobic training on parameters of physical fitness (flexibility, balance, walking speed, global physical capacity score) in adults with overweight or obesity.

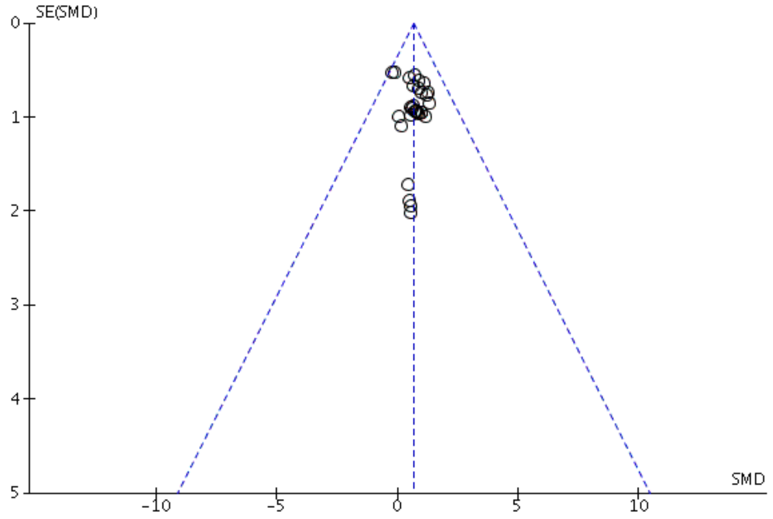

Figure S25. Funnel plot of the effect of resistance training on parameters of physical capacity in adults with overweight or obesity.
